# Alcohol Consumption and Risk of Fractures: A Systematic Review and Dose**–**Response Meta**-**Analysis of Prospective Cohort Studies

**DOI:** 10.1016/j.advnut.2023.03.008

**Published:** 2023-03-24

**Authors:** Yamin Ke, Huifang Hu, Jinli Zhang, Lijun Yuan, Tianze Li, Yifei Feng, Yuying Wu, Xueru Fu, Mengmeng Wang, Yajuan Gao, Weifeng Huo, Yaobing Chen, Wenkai Zhang, Longkang Wang, Xi Li, Jinyuan Pang, Zeqiang Zheng, Fulan Hu, Ming Zhang, Liang Sun, Yang Zhao, Jie Lu, Dongsheng Hu

**Affiliations:** 1 Department of General Practice, The Affiliated Luohu Hospital of Shenzhen University Medical School, Shenzhen, Guangdong, China, 47 Youyi Road, Luohu District, Shenzhen, Guangdong, 518001, People’s Republic of China; 2 Department of Epidemiology and Biostatistics, College of Public Health, Zhengzhou University, Zhengzhou, Henan, People’s Republic of China; 3 Department of Preventive Medicine, School of Public Health, Shenzhen University Medical School, ShenZhen, Guangdong, People’s Republic of China; 4 Department of Biostatistics and Epidemiology, School of Public Health, Shenzhen University Medical School, Shenzhen, Guangdong, People’s Republic of China; 5 Department of Social Medicine and Health Management, College of Public Health, Zhengzhou University, Zhengzhou, Henan, People’s Republic of China

**Keywords:** alcohol intake, fracture, dose**–**response, prospective cohort study, meta-analysis

## Abstract

Alcohol consumption remains inconsistently correlated with fracture risk, and a dose**–**response meta-analysis for specific outcomes is lacking. The objective of this study was to quantitatively integrate the data on the relationship between alcohol consumption and fracture risk. Pertinent articles were identified in PubMed, Web of Science, and Embase databases up to 20 February 2022. Combined RRs and 95% CIs were estimated by random- or fixed-effects models. Restricted cubic splines were used to model linear or nonlinear relationships. Forty-four articles covering 6,069,770 participants and 205,284 cases of fracture were included. The combined RRs and 95% CIs for highest compared with lowest alcohol consumption were 1.26 (1.17–1.37), 1.24 (1.13–1.35), and 1.20 (1.03–1.40) for total, osteoporotic, and hip fractures, respectively. A linear positive relationship between alcohol consumption and total fracture risk was detected (*P*_nonlinearity_ = 0.057); the risk was correlated with a 6% increase (RR, 1.06; 95% CI: 1.02, 1.10) per 14 g/d increment of alcohol consumption. J-shaped relationships of alcohol consumption with risk of osteoporotic fractures (*P*_nonlinearity_ < 0.001) and hip fractures (*P*_nonlinearity_ < 0.001) were found. Alcohol consumption of 0 to 22 g/d was linked to a reduced risk of osteoporotic fractures and hip fractures. Our findings show that any level of alcohol consumption is a risk factor for total fractures. Moreover, this dose**–**response meta-analysis shows that an alcohol consumption level of 0 to 22 g/d is related to a reduction in the risk of osteoporotic and hip fractures.

The protocol was registered in the International Prospective Register of Systematic Reviews (CRD42022320623).

**Statement of Significance**

Our study sought to quantify the dose**–**response relationship between alcohol consumption and total fractures, osteoporotic fractures, and subtypes of osteoporotic fractures (hip, wrist, and vertebral fractures). J-shaped relationships of alcohol consumption with risk of osteoporotic fractures and hip fractures were found.

## Introduction

Fractures are prevalent around the world, with the number of cases increasing to 436 million in 2019, a 69% increase since 1990 [1]. Fractures are a common public health issue, resulting in disability, chronic pain, depression, decreased quality of life, and an increased risk of premature death [[2], [3], [4], [5]]. Moreover, fractures impose an enormous burden on health care systems and societies [6,7]. In the United States, osteoporosis-related fractures result in direct medical costs of $17.9 billion per annum [8]. Preventing fractures, therefore, through identification and recognition of modifiable risk factors, is essential to public health.

Numerous factors influence the risk of fracture, including alcohol intake [[9], [10], [11], [12]], but evidence of a correlation between alcohol intake and fracture risk is inconsistent. Alcohol intake and fracture risk were found to be positively correlated in some cohort studies [[13], [14], [15]], whereas no associations or inverse associations were found in other cohort studies [[16], [17], [18]]. In addition, a previous meta-analysis covering 18 prospective cohort studies revealed a nonlinear correlation between alcohol consumption and hip fracture risk; however, that study offered no information about the association of alcohol consumption with risk of other types of fracture [19]. Recently, a meta-analysis covering 38 prospective cohort studies suggested that the consumption of alcohol was positively linked to total fracture risk, but not to the risk of specific types of fracture [20]; however, that study only conducted a binary estimate, with dose–response data lacking.

We therefore carried out a dose**–**response meta**-**analysis to explore possible linear or nonlinear relationships between alcohol consumption and risk of various types of fracture, including total fractures, osteoporotic fractures, and subtypes of osteoporotic fractures (hip, wrist, and vertebral fractures).

## Methods

### Search strategy

The protocol was registered in the International Prospective Register of Systematic Reviews (identifier: CRD42022320623). The Preferred Reporting Items for Systematic Reviews and Meta-Analyses process was followed [21].

PubMed, Web of Science, and Embase were retrieved to identify articles, from inception until February 20, 2022, that examined the correlation between alcohol consumption and fracture risk. The MeSH terms and key words utilized in this search strategy included: *alcohol; drinking behavior; ethanol; fractures, bone; fracture; osteoporotic fractures; bone mineral density; and cohort study or prospective.* Details of the search strategy appear in Supplemental Table 1. Additional eligible articles were identified by manually retrieving the citations from the searched original articles and reviews. The search retrieved only English language articles.

### Inclusion criteria

The criteria for inclusion were as follows: *1*) it was a prospective cohort study; *2*) participants were adults aged ≥ 18 at baseline; *3*) alcohol consumption was an exposure and fracture risk was an outcome; *4*) RRs or HRs or ORs and 95% CIs were provided; and *5*) for the dose**–**response analysis, alcohol consumption was given at 3 or more levels or per additional increase or other relevant data was supplied to enable calculation of alcohol consumption. If more than one article was published for the same cohort, the one with the larger number of participants or longer follow-up period or more comprehensive data was considered.

### Data extraction and quality assessment

YK and MW, 2 independent investigators, collected the following information: first author, publication year, region, study name, sample size, age of participants, follow-up years, sex, number of cases, exposure assessment, outcome, outcome assessment, adjustment for covariates, and RRs/HRs/ORs with 95% CIs for fracture risk in each alcohol consumption group. By using the Newcastle Ottawa Scale (NOS), with a highest score of 9 points and a lowest of 0, the quality of included articles was evaluated. In our study, articles with a score of 7 or higher were recognized as high quality [22]. Arbitration by a third author (DH) occurred when there were unresolved disagreements.

### Exposure harmonization

Different studies reported different units of measurement of alcohol consumption, so we transformed various units of alcohol intake into grams per day (g/d). For articles that did not specify a standard drink size, we defined each standard drink as containing 14 g of pure alcohol [23]. Since there is generally a range of alcohol consumption levels, the mid-point of the extent of alcohol consumption was estimated as exposure value. For an open-ended highest category, the upper limit was assumed to be 20% higher than the lower limit of the interval [24].

### Definition of outcome

Total fractures were defined as fractures that occurred at any site. Osteoporotic fractures were defined as sites that are age-dependent and show an association with low bone mineral density (BMD). Fractures of the skull, face, hands, fingers, feet, toes, ankle, patella, and in men, tibia and fibula were not regarded as osteoporotic fractures [25].

### Statistical methods

RRs with 95% CIs were used as the uniform effect size for studies, with HRs and ORs assumed to be approximate RRs [26]. If outcomes for osteoporotic fracture subtypes were reported in an article or in different articles in the same cohort, a fixed-effects model was used to compute RR and 95% CI, then the combined effect size was used in this analysis [27]. Any studies separately reporting results for males and females were considered as 2 independent studies. If the number of cases in each group was not offered, we used the overall number of cases and the provided RRs to calculate them [28]. The groups were deemed equal in size if the number of participants or person-years in each group was not available [28]. If effect estimates relative to moderate or other levels of alcohol intake were reported, we recalculated the RR and 95% CI by using the lowest alcohol intake as a reference [29].

To assess heterogeneity, *Q* Cochran test and *I*^2^ statistics were utilized [30]. For the *Q* statistic, *P* < 0.1 was regarded as statistically significant. *I*^2^ scores of approximately 25%, 50%, and 75% were deemed to be low, moderate, and high heterogeneity, respectively [30]. A fixed-effects model was used to estimate the combined RRs and 95% CIs when *I*^2^ was below 50% [31]; and, if not below 50%, a random-effects model was selected [27]. The study-specific dose**–**response association was estimated using generalized least-squares regression [32]. Study-specific RRs with 95% CIs for risk of fracture were calculated against each 14 g/d increment in alcohol consumption. A random- or fixed-effects model was applied to combine RRs and 95% CIs of fracture risk for high versus low levels of alcohol consumption and per 14 g/d increment [27,31]. Moreover, the nonlinear relationship was examined by modeling alcohol consumption levels with restricted cubic splines, with 3 knots at percentiles of 25, 50, and 75 of the distribution [33]. The null hypothesis test, which assumed that the coefficient of the second spline was equivalent to 0, was utilized to estimate the *P* value of nonlinearity (*P*_nonlinearity_) [26].

Subgroup analyses were investigated, stratifying for sex, age, region, sample size, follow-up years, study quality, and adjusted variables (education, BMD, fracture history, and smoking), to identify sources of heterogeneity in the study-specific analysis. *P* values for heterogeneity between subgroups were calculated by performing meta-regression analysis. In order to assess the robustness of findings, sensitivity analysis was undertaken by eliminating 1 study at each stage. Egger’s test and funnel plots were utilized to assess publication bias [34] if there were ≥ 10 studies, as recommended by the Cochrane Handbook [35]. If statistically significant publication bias was detected, the trim and fill method was utilized to correct it [36]. Stata 14.0 (Stata Corp) was utilized for analysis. All tests were 2-sided. *P* values < 0.05 were considered significant if not specifically stated.

### Certainty of evidence

The Grading of Recommendation, Assessment, Development and Evaluation (GRADE) tool was used to evaluate the quality of evidence. According to GRADE, the quality of evidence is rated at 4 levels: high, moderate, low, and very low. By default, the quality of evidence from observational studies is rated as low. Factors that can decrease the quality of evidence include risk of bias, inconsistency, indirectness, imprecision, and publication bias. Factors that can upgrade the quality of evidence include large effects, plausible confounding, and dose effect [37].

## Results

Overall, 10,500 articles were retrieved through PubMed, Web of Science, Embase, and reference lists. After excluding duplicate articles (*n* = 1,845) and reviewing titles or abstracts (*n* = 8,560), 95 articles were retrieved. Fifty-one articles were excluded (Supplemental Table 2). Finally, 44 articles (56 studies) were included in the current systematic review. Two of them did not provide sufficient information and lacked quantitative data so were only presented as a systematic review [38,39]; therefore, 42 articles (53 studies) remained in the meta-analysis [[13], [14], [15], [16], [17], [18],[40], [41], [42], [43], [44], [45], [46], [47], [48], [49], [50], [51], [52], [53], [54], [55], [56], [57], [58], [59], [60], [61], [62], [63], [64], [65], [66], [67], [68], [69], [70], [71], [72], [73], [74], [75]] (Supplemental Figure 1).

### Study characteristics

Supplemental Table 3 lists the characteristics of the 44 selected articles. Forty-four articles covered studies with 6,069,770 participants and 205,284 total cases of fracture. The sample size ranged from 181 [46] to 3,142,673 [51]. Follow-up durations varied from 1 [43,65] to 34 [17] y. Overall, 21 articles were from studies conducted in North America [15,17,18,38,43,45,48,52,[57], [58], [59],^61^,^63^,[65], [66], [67], [68],[72], [73], [74], [75]], 16 in Europe [13,14,16,39,40,42,46,[49], [50], [51],^53^,^56^,^60^,^64^,^69^,^71^], 5 in Asia [41,44,54,55,70], one each from the Netherlands, Australia, and Canada [62], and another covering 40 countries [47]. Twenty-five articles included both men and women [13,[15], [16], [17],^38^,[41], [42], [43], [44],^46^,^47^,[49], [50], [51], [52],^55^,[58], [59], [60],[62], [63], [64],^69^,^70^,^74^], 8 included only men [14,39,45,48,53,61,68,75], and 11 included only women [18,40,54,56,57,[65], [66], [67],[71], [72], [73]]. Alcohol consumption levels were generally assessed through face-to-face questionnaire interviews with food frequency questionnaires. The definition of fractures in 10 articles were based only on self-report [17,43,47,49,55,60,62,65,67,68], whereas others generally were validated by radiologic diagnoses or medical records. The average NOS score of articles was 6.89 (Supplemental Table 4).

### Findings from the systematic review

Among the studies that assessed the association between alcohol consumption and risk of total fractures, 3 studies showed an inverse association [17,42,57], and 22 indicated a significant positive association [[13], [14], [15], [16],[39], [40], [41],^44^,^46^,^48^,^50^,^51^,^53^,^55^,^59^,^62^,^63^,^67^,[69], [70], [71],^73^], whereas others did not find any significant association. In terms of osteoporotic fractures, an inverse association was found with alcohol consumption in 3 studies [17,42,57], and a significant positive association in 18 studies [15,16,40,41,44,46,48,50,51,53,55,59,62,69,70,73], but a null association in other studies. Of the 28 studies on risk of hip fractures, 3 showed an inverse association between alcohol consumption and hip fractures [17,42,57], and 10 indicated a significant association [15,16,25,50,51,53,69,70], whereas others did not show any significant association. We found 5 studies that showed a null association between alcohol and wrist fractures [38,49,66,71,75]. Of the 7 studies on vertebral fractures, 1 showed a positive association with alcohol consumption [59], whereas others did not report any significant association [49,54,64,66].

### Association of high versus low alcohol consumption with risk of fractures

**FIGURE 1:**
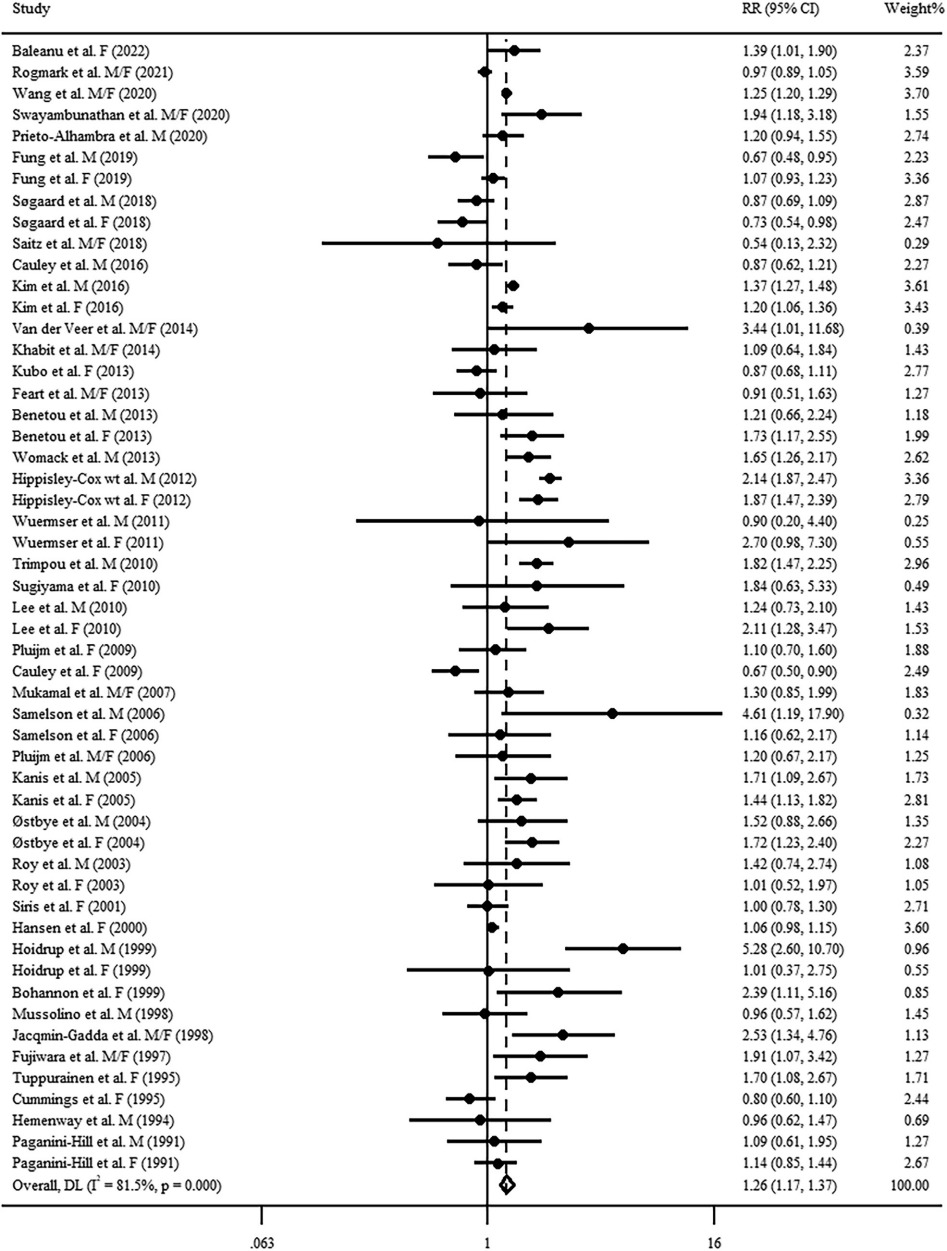
Forest plot of pooled RR for total fractures with the highest versus lowest alcohol consumption level. F, female; M, male.

**FIGURE 2:**
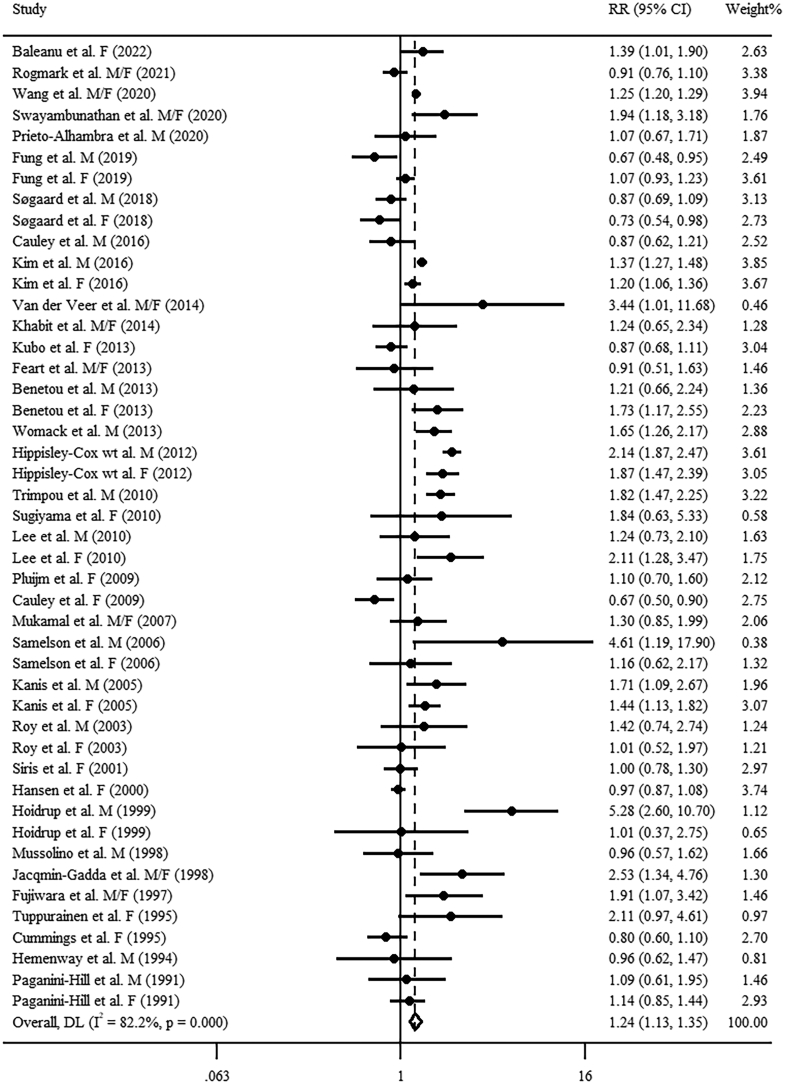
Forest plot of pooled RR for osteoporotic fractures with the highest versus lowest alcohol consumption level. F, female; M, male.

For total fractures and osteoporotic fractures, 42 articles including 53 cohort studies [[13], [14], [15], [16], [17], [18],[40], [41], [42], [43], [44], [45], [46], [47], [48], [49], [50], [51], [52], [53], [54], [55], [56], [57], [58], [59], [60], [61], [62], [63], [64], [65], [66], [67], [68], [69], [70], [71], [72], [73], [74], [75]] and 37 articles including 46 cohort studies [[13], [14], [15], [16], [17], [18],[40], [41], [42], [44], [45], [46], [47], [48], [49], [50], [51],[53], [54], [55], [56], [57], [58], [59],^61^,^62^,[64], [65], [66],[68], [69], [70], [71], [72], [73], [74], [75]] were included, respectively. Comparing the highest and lowest levels of alcohol consumption, the combined RRs for total fractures and osteoporotic fractures were 1.26 (95% CI: 1.17, 1.37; *I*^2^ = 81.5%, *P*_heterogeneity_ < 0.001) and 1.24 (95% CI: 1.13, 1.35; *I*^2^ = 82.2%, *P*_heterogeneity_ < 0.001), respectively (FIGURE 1, FIGURE 2). Egger’s test and funnel plot for both total fractures (*P* = 0.476) and osteoporotic fractures (*P* = 0.902) revealed no significant publication bias (Supplemental Figure 2A, B). Sensitivity analyses showed robust results for total fractures and osteoporotic fractures, respectively.

For hip fractures, wrist fractures, and vertebral fractures, 28 studies (21 articles) [13,[15], [16], [17], [18],^41^,^42^,^45^,[49], [50], [51],^53^,^57^,^58^,^62^,^66^,[68], [69], [70],^72^,^74^], 4 studies (4 articles) [49,66,71,75] and 7 studies (5 articles) [49,54,59,64,66] were included, respectively. Comparing alcohol consumption at the highest and the lowest levels, the combined RR was 1.20 (95% CI: 1.03, 1.40; *I*^2^ = 82.7%, *P*_heterogeneity_ < 0.001) for hip fractures, 1.04 (95% CI: 0.89, 1.20; *I*^2^ = 47.3%, *P*_heterogeneity_ = 0.127) for wrist fractures, and 1.01 (95% CI: 0.82, 1.24; *I*^2^ = 37.7%, *P*_heterogeneity_ = 0.141) for vertebral fractures (Supplemental Figures 3 and 4). Egger’s test and funnel plot for hip fractures observed no significant publication bias (*P* = 0.144, Supplemental Figure 5). The number of studies for alcohol consumption with wrist and vertebral fracture risk was too small to evaluate publication bias (*n* < 10). Sensitivity analyses indicated robust results for hip, wrist, and vertebral fractures.

### Dose**–**response relationship between alcohol consumption and risk of fractures

#### Total fractures

**FIGURE 3:**
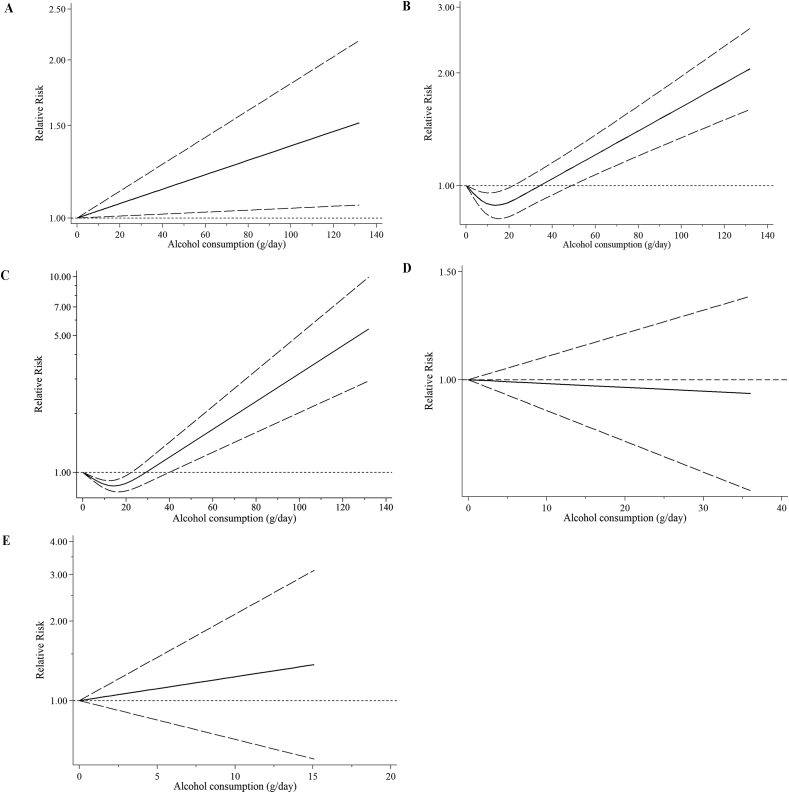
Dose**–**response association of alcohol consumption with risk of fractures by restricted cubic splines: (A) total fractures, (B) osteoporotic fractures, (C) hip fractures, (D) wrist fractures, (E) vertebral fractures.

**FIGURE 4:**
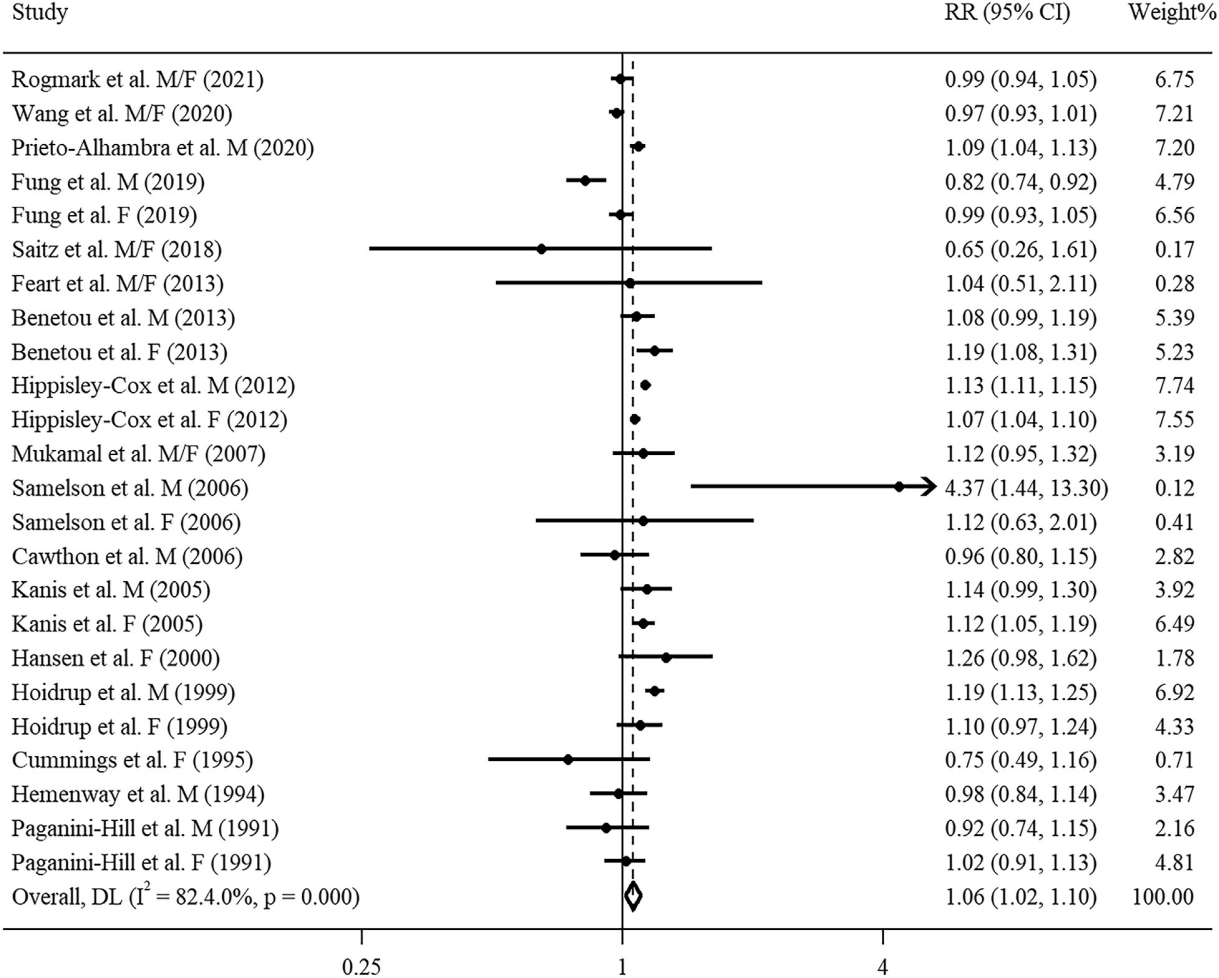
Forest plot of study-specific RR for total fractures per 14 g/d increment in alcohol consumption. F, female; M, male.

For the dose–response analysis, we included 24 studies (18 articles) [13,14,16,17,41,43,[49], [50], [51],^58^,^59^,^61^,^62^,^66^,[72], [73], [74], [75]]. A linear positive correlation of alcohol consumption with total fractures risk (*P*_nonlinearity_ = 0.057, Figure 3A) was found. The combined RR for total fractures per 14 g/d increment of alcohol consumption was 1.06 (95% CI: 1.02, 1.10; *I*^2^ = 82.4%, *P*_heterogeneity_ < 0.001, Figure 4). There was no detectable publication bias using Egger’s test and funnel plot (*P* = 0.224, Supplemental Figure 6A). Sensitivity analysis indicated robust results. Meta-regression analyses found significant heterogeneity between subgroups stratified by region (*P* = 0.031). With per 14 g/d increment in alcohol consumption, a significant effect size was observed in non-US regions (RR: 1.09; 95% CI: 1.05, 1.13; *I*^2^ = 85.3%, *P*_heterogeneity_ < 0.001,Table), but not in the United States (RR: 0.99; 95% CI: 0.90, 1.08; *I*^2^ = 59.4%, *P*_heterogeneity_ = 0.004, Table).

#### Osteoporotic fractures

**FIGURE 5:**
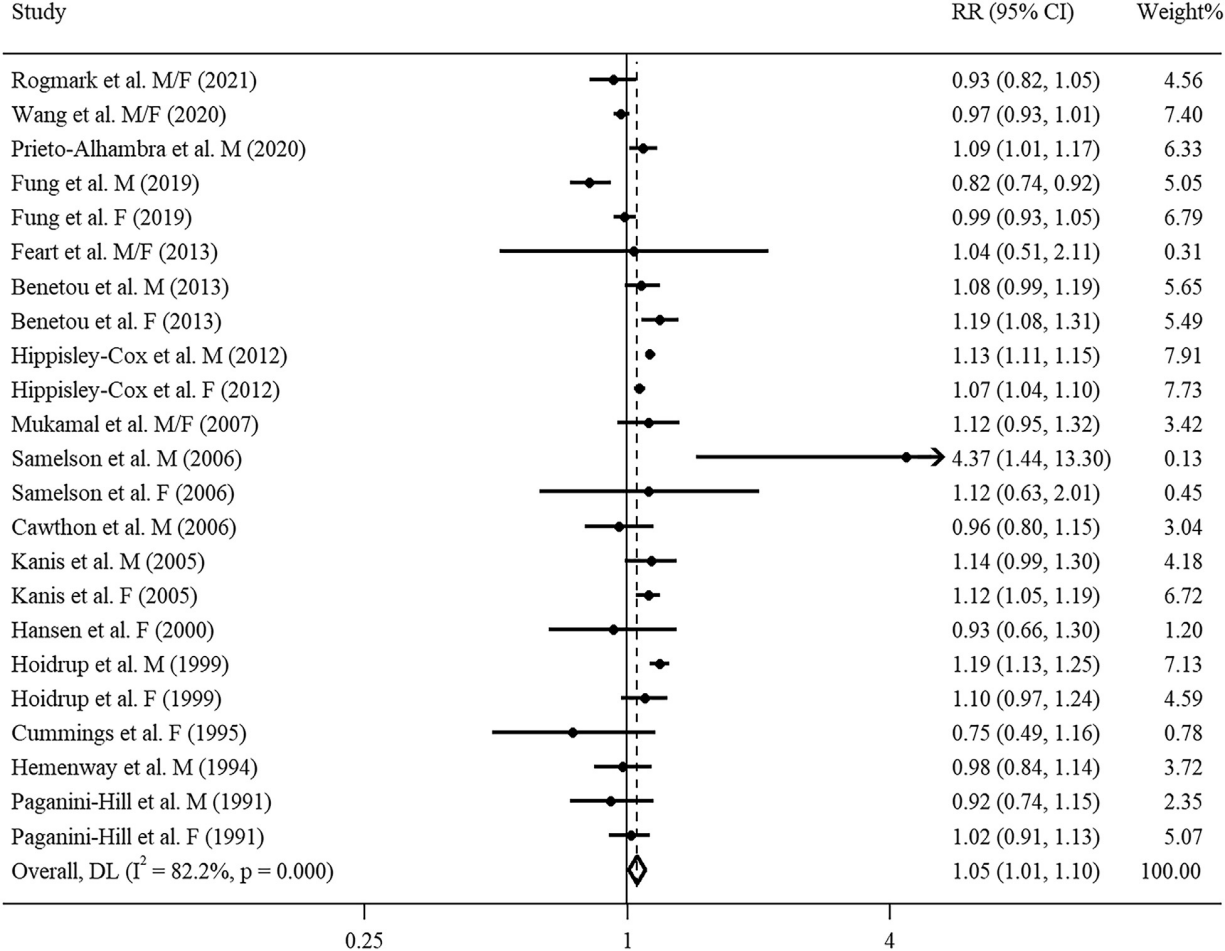
Forest plot of study-specific RR for osteoporotic fractures per 14 g/d increment in alcohol consumption. F, female; M, male.

For the dose–response analysis of alcohol consumption with osteoporotic fracture risk, based on 23 studies (17 articles) [13,14,16,17,41,[49], [50], [51],^58^,^59^,^61^,^62^,^66^,[72], [73], [74], [75]], a J-shaped relationship was observed (*P*_nonlinearity_ < 0.001, Figure 3B). Alcohol consumption of 0 to 22 g/d was related to a lower osteoporotic fracture risk, whereas the risk was significantly elevated with alcohol consumption of > 49 g/d. The combined RR for osteoporotic fractures per 14 g/d increment in alcohol consumption was 1.05 (95% CI: 1.01, 1.10; *I*^2^ = 82.2%, *P*_heterogeneity_ < 0.001, Figure 5). No detectable publication bias was observed using Egger’s test and funnel plot (*P* = 0.157, Supplemental Figure 6B). Sensitivity analysis indicated robust results. Meta-regression analyses found significant heterogeneity between subgroups stratified by region (*P* = 0.014). With per 14 g/d increment in alcohol consumption, a significant effect size was observed in non-US regions (RR: 1.09; 95% CI: 1.05, 1.14; *I*^2^ = 83.8%, *P*_heterogeneity_ < 0.001, Table), but not in the United States (RR: 0.97; 95% CI: 0.89, 1.05; *I*^2^ = 55.2%, *P*_heterogeneity_ = 0.014, Table).

#### Hip fractures

For the dose–response analysis of alcohol consumption with hip fracture risk, based on 18 studies (12 articles) [13,16,17,41,[49], [50], [51],^58^,^62^,^66^,^72^,^74^], a J-shaped relationship was observed (*P*_nonlinearity_ < 0.001, Figure 3C). Alcohol consumption of 0 to 22 g/d was correlated with a decreased hip fracture risk, whereas the risk was significantly elevated with alcohol consumption of > 40 g/d. The combined RR for hip fractures per 14 g/d increment in alcohol consumption was 1.04 (95% CI: 0.99, 1.10, *I*^2^ = 84.4%, *P*_heterogeneity_ < 0.001, Supplemental Figure 7). No detectable publication bias was observed using Egger’s test and funnel plot (*P* = 0.554, Supplemental Figure 8). Sensitivity analysis suggested that the results were robust. Meta-regression analyses found significant heterogeneity between subgroups stratified by region (*P* = 0.019). With per 14 g/d increment in alcohol consumption, a significant effect size was found in non-US regions (RR: 1.09; 95% CI: 1.03, 1.16; *I*^2^ = 85.8%, *P*_heterogeneity_ < 0.001, Supplemental Table 5), but not in the United States (RR: 0.94; 95% CI: 0.85, 1.04; *I*^2^ = 61.4%, *P*_heterogeneity_ = 0.016, Supplemental Table 5).

#### Wrist fractures

Based on 3 studies (3 articles) [49,66,75], no proof was observed for a nonlinear relationship between consumption of alcohol and risk of wrist fractures (*P*_nonlinearity_ = 0.368, Figure 3D). No association was observed for per 14 g/d increment of alcohol consumption with risk of wrist fractures (RR: 0.98; 95% CI: 0.85, 1.14, *I*^2^ =0.0%, *P*_heterogeneity_ = 0.442, Supplemental Figure 9A). Publication bias and subgroup analyses of wrist fractures were not available for evaluation because of the scarcity of studies (*n* < 10). Sensitivity analysis suggested the results were robust.

#### Vertebral fractures

Based on 4 studies (3 articles) [49,59,66], no proof was observed for a nonlinear relationship between consumption of alcohol and risk of vertebral fractures (*P*_nonlinearity_ = 0.136, Figure 3E). No association was observed for per 14 g/d increment of alcohol consumption with vertebral fracture risk (RR: 1.34; 95% CI: 0.62, 2.87, *I*^2^ = 62.7%, *P*_heterogeneity_ = 0.045, Supplemental Figure 9B). Publication bias and subgroup analyses of vertebral fractures were not available for evaluation because of the scarcity of studies (*n* < 10). Sensitivity analysis suggested the results were robust.

### Grading the evidence

Supplemental Table 6 shows the findings and quality of evidence for the association between alcohol consumption and each outcome. For total fractures and osteoporotic fractures, the certainty of evidence was moderate. For hip fractures and wrist fractures, the certainty of evidence was low due to serious imprecision. For vertebral fractures, however, the certainty of evidence was very low due to serious inconsistency and imprecision.

## Discussion

Our current meta-analysis based on 42 prospective cohort articles indicated that high alcohol consumption was linked to an elevated risk of total, osteoporotic, and hip fractures, but not for wrist and vertebral fractures. A linear positive relationship between alcohol consumption and risk of total fractures was found. With per 14 g/d increment of alcohol consumption, the total fracture risk increased by 6%. Moreover, the results found J-shaped relationships between alcohol consumption and osteoporotic and hip fractures, alcohol consumption of 0 to 22 g/d was linked to reduced risk of osteoporotic and hip fractures.

In line with our results, a recent meta-analysis by Asoudeh et al*.* [20] that included 38 prospective cohort studies suggested that high alcohol consumption was significantly linked to an elevated total fracture risk; however, this study only performed a traditional binary analysis. In our study, the dose**–**response correlation of alcohol consumption with total fractures was further quantitatively evaluated and a 6% increased risk of total fractures was found for per 14 g/d increment in alcohol consumption. Our study suggested a positive linear relationship between alcohol consumption and total fracture risk. One possible reason for this is that alcohol consumption results in an elevated risk of falls and motor vehicle accidents [61,76], possibly increasing the risk of traumatic fractures [77].

According to a meta-analysis involving 11 cohort studies by Godos et al. [78], high alcohol consumption was related to an elevated risk of osteoporotic fractures and hip fractures, a finding that aligns with our results. In comparison with this meta-analysis, we included more studies with longer follow-up durations and more individuals (6,035,122 versus 240,871), which may offer more compelling evidence for the link between alcohol consumption and osteoporotic and hip fractures. J-shaped relationships were found between the consumption of alcohol and risk of osteoporotic and hip fractures in our study. Several other meta-analyses identified similar relationships. Godos et al. [78] found a nonlinear correlation of alcohol intake with risk of osteoporotic and hip fractures, showing that intake of 3 or more drinks a day was correlated with a higher hip fracture risk. Zhang et al. [19] showed a nonlinear correlation between alcohol consumption and risk of hip fractures, suggesting that light consumption of alcohol (0.01–12.5 g/d) was linked to a decreased risk of hip fractures, whereas heavy consumption of alcohol (≥ 50 g/d) was related to an elevated risk. Similarly, our analysis demonstrated that alcohol consumption of 0 to 22 g/d was linked to a decrease in the risk of osteoporotic fractures and hip fractures, whereas alcohol consumption of > 49 g/d and > 40 g/d was correlated with a significantly elevated risk of osteoporotic and hip fractures, respectively.

Some possible mechanisms might account for the J-shaped relationship. A previous review reported that low alcohol intake may increase BMD [79]. Lower alcohol intake could promote the production of calcitonin, which suppresses bone resorption [80], and this may have a positive influence on BMD [81]. Moreover, low to moderate alcohol intake can slow age-related bone loss by reducing bone remodeling [82]. Although all of those possible mechanisms could account for the beneficial influence of alcohol on bones, chronic excessive alcohol consumption may result in a loss of bone mass and raise fracture risk [82]. First, alcohol has toxic effects on osteoblasts, which can disrupt bone remodeling by inhibiting new bone formation [83,84]. Second, alcohol intake inhibits bone formation via Wnt signaling pathways due to stimulated oxidative stress [79,85,86]. Third, alcohol can induce lipogenesis, reduce the osteogenesis of bone marrow matrix, and yield lipid deposits within cells, resulting in bone cell death [87,88]. Finally, alcohol intake may affect nutritional status and the intake of micronutrients [82], both possible pathways to malnutrition and deficiencies in calcium and vitamin D that are risk factors for bone health [[89], [90], [91]].

We also found that alcohol consumption was unrelated to the risk of wrist and vertebral fractures, which is consistent with the findings of Asoudeh et al. [20]*.* Nevertheless, these results should be regarded with caution because there was an insufficient number of studies included in these analyses. Studies on the relationship between alcohol consumption and wrist and vertebral fractures should be undertaken in the future.

To recognize possible sources of heterogeneity, different subgroup analyses and meta-regression were conducted. The heterogeneity in studies of total fractures, osteoporotic fractures, and hip fractures may have arisen from differences in the regions from which data was collected. We observed a positive relationship between alcohol intake and risk of total, osteoporotic, and hip fractures from studies conducted in non-US regions, but not from studies conducted in the United States. A possible explanation is that the types of alcohol consumed and the drinking cultures vary across geographic regions [76,92], perhaps influencing the relationship of alcohol intake and fracture risk.

Our analysis has some strengths. First, only prospective cohort studies were included so that the possibility of recall and selection bias could be minimized. Second, dose**–**response analysis was performed to model linear or nonlinear relationships. Third, the inclusion of a substantial number of subjects and cases offered high statistical power for evaluating the relationship between alcohol intake and fracture risk. Further, we analyzed subtypes of osteoporotic fractures, including hip, wrist, and vertebral fractures. A more thorough understanding of the correlation between the intake of alcohol and fracture risk was provided by these data in accordance with the current evidence.

Nevertheless, several potential limitations of this study should be acknowledged. For included studies, various methods were used to assess alcohol consumption, such as food frequency questionnaires, dietary history questionnaires, and other types of questionnaires, which may have led to measurement errors and misclassification of alcohol exposure. Second, we specified that each standard drink contained 14 g of pure alcohol for articles that did not specify a standard drink size, which may have overestimated or underestimated the actual alcohol intake. Third, since studies on the association between alcohol intake and wrist and vertebral fractures are scarce, the ability to examine correlations and to perform subgroup analysis was limited. Finally, although the analysis was controlled for potential confounders, the results may be influenced by residual or unmeasured confounders.

**TABLE:** Subgroup analyses of dose**–**response risk of total fractures and osteoporotic fractures with alcohol consumption

Subgroup	Total fractures (per 14 g/d increment)				
	No. of studies	RR (95% CI)	*I*^*2*^(%)	*P*^1^	*P*^2^
All studies	24	1.06 (1.02, 1.10)	82.4	< 0.001	
**Sex**					0.583
Male	10	1.06 (1.00, 1.13)	83.0	< 0.001	
Female	9	1.08 (1.03, 1.13)	56.1	0.020	
Both	5	0.98 (0.95, 1.01)	0.0	0.449	
**Age**					0.714
< 60	14	1.07 (1.02, 1.11)	84.9	< 0.001	
≥ 60	10	1.04 (0.97, 1.12)	62.5	0.004	
**Region**					0.031
US	12	0.99 (0.90, 1.08)	59.4	0.004	
Non-US	12	1.09 (1.05, 1.13)	85.3	< 0.001	
**Sample size**					0.702
< 10,000	7	1.02 (0.83, 1.25)	46.5	0.082	
≥ 10,000	17	1.06 (1.02, 1.10)	86.4	< 0.001	
**Follow-up years**					0.362
< 8 y	10	1.03 (0.96, 1.11)	83.4	0.005	
≥ 8 y	13	1.08 (1.03, 1.13)	62.3	< 0.001	
**Study quality**					0.147
high	21	1.06 (1.02, 1.11)	83.8	< 0.001	
medium	3	0.91 (0.78, 1.09)	-	0.443	
**Adjustment**					
**Education**					
Yes	7	1.10 (1.04, 1.17)	77.1	< 0.001	0.182
No	17	1.03 (0.98, 1.09)	84.4	< 0.001	
**BMD**					0.356
Yes	2	1.12 (1.06, 1.19)	-	0.817	
No	22	1.05 (1.01, 1.09)	83.6	< 0.001	
**Fracture history**					0.836
Yes	4	1.04 (0.96, 1.13)	82.6	0.001	
No	20	1.06 (1.02, 1.11)	76.5	< 0.001	
**Smoking**					0.130
Yes	20	1.07 (1.03, 1.12)	83.3	< 0.001	
No	3	0.99 (0.91, 1.09)	0.0	0.368	

Subgroup	Osteoporotic fractures (per 14 g/d increment)				
	No. of studies	RR (95% CI)	*I*^*2*^(%)	*P*^1^	*P*^2^
All studies	23	1.05 (1.01, 1.10)	82.2	< 0.001	
**Sex**					0.504
Male	10	1.06 (0.99, 1.13)	82.6	< 0.001	
Female	9	1.07 (1.02, 1.12)	53.6	0.028	
Both	4	0.98 (0.93, 1.03)	11.2	0.337	
**Age**					0.515
< 60	13	1.06 (1.01, 1.12)	84.6	< 0.001	
≥ 60	10	1.03 (0.96, 1.10)	58.3	0.01	
**Region**					0.014
US	11	0.97 (0.89, 1.05)	55.2	0.014	
Non-US	12	1.09 (1.05, 1.14)	83.8	< 0.001	
**Sample size**					0.886
< 10,000	6	1.05 (0.84, 1.29)	51.0	0.070	
≥ 10,000	17	1.05 (1.01, 1.10)	85.7	< 0.001	
**Follow-up years**					0.261
< 8 y	9	1.02 (0.95, 1.09)	60.7	0.009	
≥ 8 y	13	1.08 (1.03, 1.13)	81.2	< 0.001	
**Study quality**					0.226
high	21	1.06 (1.01, 1.10)	83.0	< 0.001	
medium	2	0.92 (0.77, 1.11)	6.6	0.301	
**Adjustment**					
**Education**					0.163
Yes	7	1.10 (1.03, 1.18)	64.2	0.010	
No	16	1.02 (0.97, 1.08)	85.1	< 0.001	
**BMD**					0.310
Yes	2	1.12 (1.06, 1.19)	-	0.817	
No	21	1.04 (1.00, 1.09)	83.5	< 0.001	
**Fracture history**					0.828
Yes	4	1.04 (0.94, 1.15)	83.8	< 0.001	
No	19	1.06 (1.01, 1.10)	77.3	< 0.001	
**Smoking**					0.157
Yes	19	1.06 (1.02, 1.11)	84.2	< 0.001	
No	4	0.98 (0.90, 1.07)	0.0	0.499	

BMD, bone mineral density.

*P*^1^: *P* value for heterogeneity within each subgroup. *P*^2^: *P* value for heterogeneity between subgroups with meta-regression analysis.

In conclusion, this study indicates that the consumption of alcohol is positively related to the risk of total, osteoporotic, and hip fractures. Overall, for total fractures, the linear relationship suggests that any level of alcohol consumption is a risk factor. Moreover, this dose**–**response meta-analysis shows that an alcohol consumption of 0 to 22 g/d is related to a reduction in the risk of osteoporotic and hip fractures. More large prospective cohort studies should be conducted to clarify the relationship between alcohol consumption and wrist and vertebral fractures.

## Data Availability

Data are available upon reasonable request.
