# Supplementary material for: Alcohol Consumption and Risk of Fractures: A Systematic Review and Dose–Response Meta-Analysis of Prospective Cohort Studies
Source: Adv Nutr. 2023 Mar 24;14(4):599–611. doi: 10.1016/j.advnut.2023.03.008 (PMC10334160; doi:10.1016/j.advnut.2023.03.008)
Supplement: Multimedia component 1 [file mmc1.docx]

**Title: Alcohol consumption and risk of fractures: a systematic review and dose–response meta-analysis of prospective cohort studies**

**First author:**

Yamin Ke^1^

**Institutional address:**

^1^Department of General Practice, The Affiliated Luohu Hospital of Shenzhen University Medical School, Shenzhen, Guangdong, China, 47 Youyi Road, Luohu District, Shenzhen, Guangdong, 518001, People’s Republic of China

**Online Supplemental Materials**

**Supplemental Table legend:**

**Supplemental Table 1** Systematic literature review search terms and strategy

**Supplemental Table 2** List of excluded studies and exclusion reason

**Supplemental Table 3** Summary of the characteristics of prospective cohort studies included in this study

**Supplemental Table 4** Quality assessment of included cohort studies (Newcastle-Ottawa Scale)

**Supplemental Table 5** Subgroup analyses of dose–response risk of hip fractures with alcohol consumption

**Supplemental Table 6** GRADE evidence table for the association of alcohol consumption with the risk of fractures

**Supplemental Figure legend:**

**Supplemental Figure 1** Flow–chart of article selection

**Supplemental Figure 2** Funnel plot for publication bias for risk of fractures with highest versus lowest alcohol consumption level (A: total fractures, B: osteoporotic fractures)

**Supplemental Figure 3** Forest plot of pooled relative risk for hip fractures with the highest versus lowest alcohol consumption level (CI, confidence interval; F, female; M, male; RR, relative risk)

**Supplemental Figure 4** Forest plot of pooled relative risks for wrist fractures (A) and vertebral fractures (B) with the highest versus lowest alcohol consumption level (CI, confidence interval; F, female; M, male; RR, relative risk)

**Supplemental Figure 5** Funnel plot for publication bias for risk of hip fractures with highest versus lowest alcohol consumption level

**Supplemental Figure 6** Funnel plot for publication bias for risk of fractures with per 14 g/d increment in alcohol consumption (A: total fractures, B: osteoporotic fractures)

**Supplemental Figure 7** Forest plot of study-specific relative risk for hip fractures per 14 g/d increment in alcohol consumption (CI, confidence interval; F, female; M, male; RR, relative risk)

**Supplemental Figure 8** Funnel plot for publication bias for risk of hip fractures with per 14 g/d increment in alcohol consumption

**Supplemental Figure 9** Forest plot of study-specific relative risks for wrist fractures (A) and vertebral fractures (B) per 14 g/d increment in alcohol consumption. (CI, confidence interval; F, female; M, male; RR, relative risk)

**Supplemental Table 1** Systematic literature review search terms and strategy

| **Search terms for PubMed（2707）** |
| --- |
| #1 (“alcohols” [Mesh] OR “ethanol” [Mesh] OR “Drinking Behavior” [Mesh] OR “alcohol drinking” [Mesh] OR “Beer” [Mesh] OR alcohol [Title/Abstract] OR ethanol [Title/Abstract] OR “wine” [Mesh] OR “alcohol drinking” [Title/Abstract] OR “alcoholic beverages” [Title/Abstract] OR “Drinking Behavior” [Title/Abstract] OR “ethanol intake” [Title/Abstract] OR “wine” [Title/Abstract] OR “Beer” [Title/Abstract] OR “liquor” [Title/Abstract] OR “spirit” [Title/Abstract]) |
| #2 ("fractures, bone"[Mesh] OR "fractures"[Title/Abstract] OR "bone"[Title/Abstract] OR "bone fractures"[Title/Abstract] OR "fracture"[Title/Abstract] OR "bone fracture"[Title/Abstract] OR "osteoporotic fracture"[Title/Abstract] OR "broken bone"[Title/Abstract] OR "bone mineral density"[Title/Abstract] OR "BMD"[Title/Abstract] OR "bone mass density"[Title/Abstract] OR "osteoporosis"[Title/Abstract] OR "bone health"[Title/Abstract] OR "osteoporosis postmenopausal"[Title/Abstract] OR "fracture bone"[Title/Abstract] OR "bone density"[Title/Abstract] OR "bone mineral content"[Title/Abstract]) |
| #3 ("Cohort Studies"[Mesh] OR "follow-up"[Title/Abstract] OR "longitudinal"[Title/Abstract] OR "cohort"[Title/Abstract] OR "prospective"[Title/Abstract]) |
| #1 AND #2 AND #3 |
| **Search terms for Embase（4520）** |
| #1 alcohol/ OR dinking behavior/ OR beer/ OR alcohol consumption/ OR wine/ OR red wine/ OR white wine/ OR alcoholic beverages/ OR liquor/ OR alcohol.mp. OR dinking behavior.mp. OR beer.mp. OR alcohol consumption.mp. OR wine.mp. OR red wine.mp. OR white wine.mp. OR alcoholic beverages.mp. OR liquor.mp. OR alcohols.mp. OR ethanol.mp. OR alcohol drinking.mp. OR ethanol intake.mp. OR spirit.mp. |
| #2 fracture/ OR bone/ OR bone density/ OR bone fragility/ OR bone mineral/ OR fragility bone/ OR bone mass/ OR osteoporosis/ OR postmenopausal osteoporosis/ OR senile osteoporosis/ OR fracture.mp. OR bone.mp. OR bone density.mp. OR bone fragility.mp. OR bone mineral.mp. OR fragility bone.mp. OR bone mass.mp. OR osteoporosis.mp. OR postmenopausal osteoporosis.mp. OR senile osteoporosis.mp. OR fractures, bone.mp. OR fractures.mp. OR bone fractures.mp. OR bone fracture.mp. OR osteoporotic fracture.mp. OR broken bone.mp. OR bone mineral density.mp. OR BMD.mp. OR bone mass density.mp. OR bone health.mp. OR osteoporosis postmenopausal.mp. OR fracture bone.mp. OR bone mineral content.mp. |
| #3 cohort analysis/ OR longitudinal study/ OR prospective study/ OR follow up/ OR prospective.mp. OR longitudinal.mp. OR cohort.mp. OR follow-up.mp. OR followed up.mp. |
| #1 AND #2 AND #3 |
| **Search terms for Web of Science（3270）** |
| #1 ("alcohols" OR "ethanol" OR "Drinking Behavior" OR "alcohol drinking" OR "Beer" OR "alcohol" OR "wine" OR "alcoholic beverages" OR "ethanol intake" OR "liquor" OR "spirit") |
| #2 ("fractures, bone" OR "fractures" OR "bone" OR "bone fractures" OR "fracture" OR "bone fracture" OR "osteoporotic fracture" OR "broken bone" OR "bone mineral density" OR "BMD" OR "bone mass density" OR "osteoporosis" OR "bone health" OR "osteoporosis postmenopausal" OR "fracture bone" OR "bone density" OR "bone mineral content") |
| #3 ("Cohort Studies" OR "follow-up" OR "longitudinal" OR "cohort" OR "prospective") |
| #1 AND #2 AND #3 |

**Supplemental Table 2.** List of excluded studies and exclusion reason

| Exclusion reason | Reference number |
| --- | --- |
| Exposure or outcome of no interest (n=17) | [1-17] |
| Not usable results (n=9) | [18-26] |
| Duplicate cohort studies (n=4) | [27-30] |
| Not-prospective cohort studies (n=3) | [31-33] |
| Reviews and meta-analysis (n=7) | [34-40] |
| Conference articles or abstracts (n=11) | [41-51] |

**References:**

1. Bailey RL, Zou P, Wallace TC, McCabe GP, Craig BA, Jun S, et al. Calcium Supplement Use Is Associated With Less Bone Mineral Density Loss, But Does Not Lessen the Risk of Bone Fracture Across the Menopause Transition: Data From the Study of Women's Health Across the Nation. JBMR Plus 2020;4(1) (no pagination)

2. Caire-Juvera G, Ritenbaugh C, Wactawski-Wende J, Snetselaar LG, Chen Z. Vitamin A and retinol intakes and the risk of fractures among participants of the Women's Health Initiative Observational Study. Am J Clin Nutr 2009;89(1):323-30.

3. Huopio J, Kroger H, Honkanen R, Saarikoski S, Alhava E. Risk factors for perimenopausal fractures: A prospective study. Osteoporosis International 2000;11(3):219-27.

4. Meyer HE, Tverdal A, Falch JA. RISK-FACTORS FOR HIP FRACTURE IN MIDDLE-AGED NORWEGIAN WOMEN AND MEN. American Journal of Epidemiology 1993;137(11):1203-11.

5. Shieh A, Ruppert KM, Greendale GA, Lian Y, Cauley JA, Burnett-Bowie SA, et al. Associations of age at menopause with postmenopausal bone mineral density and fracture risk in women. The Journal of clinical endocrinology and metabolism 2021;19

6. Tiihonen R, Paattiniemi E-L, Nurmi-Luthje I, Kaukonen J-P, Sarkkinen H, Luthje P. No change in the use of alcohol among hip fracture patients over a 12-year period: a prospective study in south-eastern Finland. Acta Orthopaedica Belgica 2021;87(ES1):89-97.

7. Burger H, de Laet CE, van Daele PL, Weel AE, Witteman JC, Hofman A, et al. Risk factors for increased bone loss in an elderly population: the Rotterdam Study. Am J Epidemiol 1998;147(9):871-9.

8. Cho Y, Choi S, Kim K, Lee G, Park SM. Association between alcohol consumption and bone mineral density in elderly Korean men and women. Archives of Osteoporosis 2018;13(1) (no pagination)

9. Felson DT, Zhang Y, Hannan MT, Kannel WB, Kiel DP. Alcohol intake and bone mineral density in elderly men and women: The Framingham study. American Journal of Epidemiology 1995;142(5):485-92.

10. Feskanich D, Korrick SA, Greenspan SL, Rosen HN, Colditz GA. Moderate alcohol consumption and bone density among postmenopausal women. Journal of Women's Health 1999;8(1):65-73.

11. Hansen MA, Overgaard K, Riis BJ, Christiansen C. Potential risk factors for development of postmenopausal osteoporosis--examined over a 12-year period. Osteoporosis international: a journal established as result of cooperation between the European Foundation for Osteoporosis and the National Osteoporosis Foundation of the USA 1991;1(2):95-102.

12. Hirata H, Kitamura K, Saito T, Kobayashi R, Iwasaki M, Yoshihara A, et al. Association between dietary intake and bone mineral density in Japanese postmenopausal women: The yokogoshi cohort study. Tohoku Journal of Experimental Medicine 2016;239(2):95-101.

13. Nguyen TV, Kelly PJ, Sambrook PN, Gilbert C, Pocock NA, Eisman JA. Lifestyle factors and bone density in the elderly: Implications for osteoporosis prevention. Journal of Bone and Mineral Research 1994;9(9):1339-46.

14. Paccou J, Edwards MH, Ward K, Jameson K, Moon R, Dennison E, et al. Relationships between bone geometry, volumetric bone mineral density and bone microarchitecture of the distal radius and tibia with alcohol consumption. Bone 2015;78:122-9.

15. Scholes S, Panesar S, Shelton NJ, Francis RM, Mirza S, Mindell JS, et al. Epidemiology of lifetime fracture prevalence in England: a population study of adults aged 55 years and over. Age and Ageing 2014;43(2):234-40.

16. Sommer I, Erkkila AT, Jarvinen R, Mursu J, Sirola J, Jurvelin JS, et al. Alcohol consumption and bone mineral density in elderly women. Public health nutrition 2013;16(4):704-12.

17. Tucker KL, Jugdaohsingh R, Powell JJ, Qiao N, Hannan MT, Sripanyakorn S, et al. Effects of beer, wine, and liquor intakes on bone mineral density in older men and women. American Journal of Clinical Nutrition 2009;89(4):1188-96.

18. Albrand G, Munoz F, Sornay-Rendu E, DuBoeuf F, Delmas PD. Independent predictors of all osteoporosis-related fractures in healthy postmenopausal women: The OFELY Study. Bone 2003;32(1):78-85.

19. Byberg L, Bellavia A, Larsson SC, Orsini N, Wolk A, Michaelsson K. Mediterranean Diet and Hip Fracture in Swedish Men and Women. Journal of Bone and Mineral Research 2016;31(12):2098-105.

20. Dhibar D, Gogate Y, Aggarwal S, Garg S, Bhansali A, Bhadada S. Predictors and outcome of fragility hip fracture: A prospective study from North India. Indian Journal of Endocrinology and Metabolism 2019;23(3):282-8.

21. Francesco L, Elisa B, Raffaella M, Alessandro P, Iacopo C, Giampiero M, et al. Assessing Risk of Osteoporotic Fractures in Primary Care: Development and Validation of the FRA-HS Algorithm. Calcified Tissue International 2017;100(6):537-49.

22. Huang Z, Himes JH, McGovern PG. Nutrition and subsequent hip fracture risk among a national cohort of white women. American Journal of Epidemiology 1996;144(2):124-34.

23. Kauppi M, Heliovaara M, Impivaara O, Knekt P, Jula A. Parity and risk of hip fracture in postmenopausal women. Osteoporosis International 2011;22(6):1765-71.

24. Pasco JA, Anderson KB, Hyde NK, Williams LJ, Rufus-Membere P, Holloway-Kew KL. High alcohol intake in older men and the probability of osteoporotic fracture according to the FRAX algorithm. Nutrients 2021;13(9) (no pagination)

25. Samieri C, Ginder Coupez V, Lorrain S, Letenneur L, Alles B, Feart C, et al. Nutrient patterns and risk of fracture in older subjects: Results from the Three-City Study. Osteoporosis International 2013;24(4):1295-305.

26. Yin J, Winzenberg T, Quinn S, Giles G, Jones G. Beverage-specific alcohol intake and bone loss in older men and women: A longitudinal study. European Journal of Clinical Nutrition 2011;65(4):526-32.

27. Benetou V, Orfanos P, Zylis D, Sieri S, Contiero P, Tumino R, et al. Diet and hip fractures among elderly Europeans in the EPIC cohort. Eur J Clin Nutr 2011;65(1):132-9.

28. Hemenway D, Colditz GA, Willett WC, Stampfer MJ, Speizer FE. Fractures and lifestyle: Effect of cigarette smoking, alcohol intake, and relative weight on the risk of hip and forearm fractures in middle-aged women. American Journal of Public Health 1988;78(12):1554-8.

29. Høidrup S, Grønbaek M, Pedersen AT, Lauritzen JB, Gottschau A, Schroll M. Hormone replacement therapy and hip fracture risk: effect modification by tobacco smoking, alcohol intake, physical activity, and body mass index. Am J Epidemiol 1999;150(10):1085-93.

30. Nguyen TV, Eisman JA, Kelly PJ, Sambrook PN. Risk factors for osteoporotic fractures in elderly men. American Journal of Epidemiology 1996;144(3):255-63.

31. Felson DT, Kiel DP, Anderson JJ, Kannel WB. Alcohol consumption and hip fractures: The Framingham Study. American Journal of Epidemiology 1988;128(5):1102-10.

32. Hino S, Yamada M, Iijima Y, Araki R, Kaneko T, Horie N. Effects of alcohol consumption on maxillofacial fractures in simple falls. Clinical and experimental dental research 2020;6(5):544-9.

33. Lin CC, Li CI, Liu CS, Wang MC, Lin CH, Lin WY, et al. Lifetime risks of hip fracture in patients with type 2 diabetic: Taiwan Diabetes Study. Osteoporosis International 2021;

34. Berg KM, Kunins HV, Jackson JL, Nahvi S, Chaudhry A, Harris KA, et al. Association between alcohol consumption and both osteoporotic fracture and bone density. American Journal of Medicine 2008;121(5):406-18.

35. Davison KS, Kendler DL, Ammann P, Bauer DC, Dempster DW, Dian L, et al. Assessing Fracture Risk and Effects of Osteoporosis Drugs: Bone Mineral Density and Beyond. American Journal of Medicine 2009;122(11):992-7.

36. Kuller LH, Meilahn EN, Cauley JA, Gutai JP, Matthews KA. Epidemiologic studies of menopause: Changes in risk factors and disease. Experimental Gerontology 1994;29(3-4):495-509.

37. Mostofsky E, Mukamal KJ, Giovannucci EL, Stampfer MJ, Rimm EB. Key Findings on Alcohol Consumption and a Variety of Health Outcomes From the Nurses' Health Study. American journal of public health 2016;106(9):1586-91.

38. Scott JC. OSTEOPOROSIS AND HIP-FRACTURES. Rheumatic Disease Clinics of North America 1990;16(3):717-40.

39. Wilsnack RW, Wilsnack SC. Alcohol use and menopause. Menopause-the Journal of the North American Menopause Society 2016;23(4):458-60.

40. Zhang X, Yu Z, Yu M, Qu X. Alcohol consumption and hip fracture risk. Osteoporosis International 2015;26(2):531-42.

41. Collin F, Duval X, Le Moing V, Piroth L, Al Kaied F, Massip P, et al. Ten-year incidence and risk factors of bone fractures in a cohort of treated HIV1-infected adults. Aids 2009;23(8):1021-4.

42. Gonzalez-Macias J, Diez-Perez A, Vilac J, Marin F. Osteoporitic fracture risk factors in Spain. [Spanish]. Medicina Clinica 2008;130(13):517.

43. Holbrook TL, Wingard DL, Barrettconnor E. DIETARY CALCIUM, CAFFEINE, AND ALCOHOL, AND RISK OF HIP FRACTURE - A 15-YEAR PROSPECTIVE-STUDY. American Journal of Epidemiology 1988;128(4):902-3.

44. Hooven FH, Boonen S, Adami S, Chapurlat R, Compston JE, Cooper C, et al. Risk factor characteristics of women with incident fractures: The global longitudinal study of osteoporosis in women. Osteoporosis International 2010;1):S99-S100.

45. Huang M, Palermo L, Stone K, Cummings SR, Kado DM. Hyperkyphosis and physical function in older community dwelling women: The study of osteoporostic fractures (SOF). Journal of Bone and Mineral Research 2007;22:S200-S1.

46. Mawondo M, Dharia S, Campbell L, Jha P. Relationship of bone mineral density and fragility fracture: An experience from a community bone service. Osteoporosis International 2010;3):S505.

47. Moayyeri A, Luben RN, Bingham S, Wareham NJ, Kaptoge S, Khaw K. Absolute risk of fractures in middle-aged and older men and women: The European prospective investigation into Cancer-Norfolk study. Calcified Tissue International 2008;82:S175-S.

48. Omsland TK, Ahmed L, Gonskag A, Schei B, Emaus N, Langhammer A, et al. Urban-rural differences in forearm fractures in postmenopausal women - The norepos study. Osteoporosis International 2010;1):S258-S9.

49. Salonen AV, Honkanen RJ, Kroger HP, Tuppurainen M. Moderate use of alcohol prevents bone loss in postmenopausal women - A population-based prospective cohort study. Journal of Bone and Mineral Research 2000;15:S416-S.

50. Yin J, Winzenberg T, Quinn S, Jones G. Drink protects BMD and decreases fracture risk in elderly. Bone 2009;1):S119.

51. Zhang J, Jameson K, Sayer AA, Robinson S, Cooper C, Dennison E. Clustering of lifestyle risk factors and low bone density in older adults: The hertfordshire cohort study. Rheumatology (United Kingdom) 2015;54(Supplement 1):i66.

**Supplemental Table 3** Summary of the characteristics of prospective cohort studies included in this study

| **Author, publication year (region)** | **Study name** | **Sample size** | **Age (years), mean or range** | **Follow-up years** | **Sex** | **Cases** | **Outcome** | **Exposure assessment** | **Outcome assessment** | **Adjustment** |
| --- | --- | --- | --- | --- | --- | --- | --- | --- | --- | --- |
| Baleanu et al., 2022 (Belgium) | Fracture Risk Brussels Epidemiological Enquiry (FRISBEE) | 3,560 | 60-85 | 5 | F | 304 | Osteoporotic fractures | NA | validated by radiographs or by a surgical report | age, body mass index, history of fracture and fall, corticosteroids use, sedentary lifestyle, sleep disturbances, rheumatoid arthritis, education, smoking, early non-substituted menopause, osteoporosis, osteopenia |
| Rogmark et al., 2021 (Sweden) | Malmö Diet and Cancer (MDC) study | 30,446 | 58 | 20.7 | M/F | 8,240 | Any fractures | Questionnaire | ICD9, ICD10 | age, sex, body mass index, previous fracture, reported family history of fracture >50 years, highest education, low leisure-time physical activity, no heavy work, living alone, feeling of loneliness, number of friends, recent mental stress, current smoking |
|  |  |  |  |  |  | 1,600 | Hip fractures |  |  |  |
| Wang et al., 2020 (Korea) | National Health  Insurance Service-Health Screening Cohort (NHIS-HEALS) | 1,431,539 | 66 | 3.52±1.76 | M/F | 87,229 | Any fractures | Questionnaire | ICD-10 | sex, income, diabetes, hypertension, and hyperlipidemia, smoking, physical exercise, body mass index, fracture history |
|  |  |  |  |  |  | 4,792 | Hip fractures |  |  |  |
| Swayambunathan et al., 2020  (USA) | Framingham Heart Study | 10,552 | ≥60 | 40 | M/F | NA | Hip fractures | Questionnaire | Self-report, validated by medical records | age, sex, diabetes, smoking, obesity, underweight, early menopause |
| Prieto-Alhambra et al., 2020 (Sweden) | NA | 36,060 | 18 | 24 | M | 3,571 | Any fractures | Questionnaire | ICD-9, ICD-10 | weight, height, parental education, and smoking |
|  |  |  |  |  |  | 1,013 | Osteoporotic fractures |  |  |  |
| Fung et al., 2019  (USA) | Health Professionals Follow-Up Study/Nurses’ Health Study | 113,578 | ≥50 | ≤34 | M/F | 3,069 | Hip fractures | FFQ | Self-report | age, body mass index, height, smoking, physical activity, energy intake, multivitamin use, caffeine, sugar–sweetened beverages, thiazide use, protein, history of diabetes, postmenopausal hormone use (women only), total intake of vitamin K, retinol, vitamin D, and calcium |
| Wright et al., 2018 (USA) | Osteoporotic Fractures in Men (MrOS) study | 5,875 | ≥65 | 10.8 | M | 94 | Wrist fractures | Questionnaire | Medical records and radiology reports | age, race/ethnicity, weight, height, body mass index, self-reported overall health, alcohol use, tobacco use, measured BMD, fall and fracture history, serum phosphate, serum calcium, estimated glomerular filtration rate (eGFR), comorbidities, physical activity, functional status, prescription medication use, calcium and vitamin D intake, and physical performance tests (including chair stands, narrow walk, grip strength, leg power, and walking speed) |
| Søgaard et al., 2018 (Norway) | Cohort of Norway | 141,925 | ≥30 | 15 | M/F | 4,831 | Hip fractures | Questionnaire | surgical procedure codes, additional diagnosis codes, and time between hospitalizations | age, height, body mass index, smoking, physical activity, self-perceived health, place of study |
| Saitz et al., 2018  (USA) | Boston ARCH prospective cohort study | 234 | 50 | 1 | M/F | 17 | Any fractures | Interview | Self-report | age, sex, race/ethnicity, duration of HIV infection, NIAAA drinking group, DXA machine used, lifetime drinking volume, years of regular cocaine use, tenofovir use, menopause, ever injection drug use, body mass index, current smoker, CD4 cell count, calcium, weight bearing physical activity, total 25(OH)D ng/mL, recent cocaine use, recent opioid use, and current ART use |
| Kim et al., 2016  (Korea) | Korean National Health Insurance Service | 359,253 | 50-90 | 7 | M/F | 20,972 | Osteoporotic fractures | Questionnaire | ICD-10 | age, body mass index, recent fragility fracture, current smoking, exercise, recent use of oral glucocorticoids and other causes of secondary osteoporosis |
| Cauley et al., 2016 (USA) | Osteoporotic Fractures in Men (MrOS) study | 5,876 | >65 | 8.6 | M | 178 | Hip fractures | FFQ | Medical records and self-report | age, race, clinic, BMD |
| Van der Veer et al., 2014 (The Netherlands) | NA | 181 | 46 | 5.4 | M/F | 56 | Fragility fractures | Questionnaire | Medical records, radiographs and questionnaires | age, sex, sCTX Z-score≥11.0, Hip BMD T-score≦21.0, UP absence |
| Khabit et al., 2014 (40 countries) | NA | 26,335 | 66.5 | 5.6 | M/F | 1,079 | Any fractures | Questionnaire | Self-report | age, sex, body mass index, region, diet quality, medical history of coronary artery disease, stroke or transient ischemic attack, hypertension, diabetes, use of statins, hormone replacement therapy and treatment allocation |
|  |  |  |  |  |  | 749 | Osteoporotic fractures |  |  |  |
| Kubo et al., 2013  (USA) | Women’s Health Initiative (WHI) | 115,655 | 63.1 | 3 | F | 1,290 | Hip fractures | FFQ | Medical records | beer consumption, liquor consumption, wine consumption, ethnicity, age group, education, osteoporosis, falls in the past year, smoking status, bisphosphonate drug use, HT status, physical activity measured in MET hours, body mass index category, HT trial arm, CaD trial arm, OS versus CT cohort, previous hip fracture at age 55+, and parental history of hip fracture at age 40+ |
| Feart et al., 2013  (France) | Three-City study | 1,435 | 75.9  (67.7-94.9) | 8 | M/F | 173 | Osteoporotic fractures | FFQ | Self-report | age, sex, physical activity and total energy intake, each individual food group component of the Mediterranean diet score, educational level, marital status, smoking status, body mass index, self-reported osteoporosis, osteoporosis treatment, calcium and/or vitamin D treatment |
|  |  |  |  |  |  | 57 | Hip fractures |  |  |  |
|  |  |  |  |  |  | 43 | Vertebral fractures |  |  |  |
|  |  |  |  |  |  | 73 | Wrist fractures |  |  |  |
| Benetou et al., 2013 (8 European countries*) | European Prospective Investigation into Cancer and nutrition study | 188,795 | 48.6 (35-70) | 9 | M/F | 802 | Hip fractures | FFQ, DHQ | Active follow-up methods, record linkage with hospital discharge records, registries | age, sex, education, smoking status, body mass index, height, physical activity, total energy intake from calibrated data, history of cardiovascular disease, history of cancer, history of fracture, menopausal status and use of hormones in a combined variable |
| Womack et al., 2013 (USA) | Veterans Aging Cohort Study (VACS) | 40,115 | 46 | 6 | M | 588 | Fragility fractures | NA | ICD-9, validated by medical records | age, race/ethnicity, body mass index, past or current smoking, inhaled/oral corticosteroid use, proton pump inhibitor use, comorbid conditions and VACS Index score |
| Hippisley-Cox et al., 2012 (UK) | NA | 3,142,673 | 50(30-100) | 10 | M/F | 59,772 | Osteoporotic fractures | Person’s electronic healthcare record | General practice records or linked cause of death records | Ethnic origin, smoking status, and medical and social factors |
|  |  |  |  |  |  | 20,028 | Hip fractures |  |  |  |
| Wuermser et al., 2011 (USA) | NA | 699 | 21-93 | 13.9 | M/F | 56 | Rib fractures | Interview | Periodic interview and medical record review | age |
| Trimpou et al., 2010 (Sweden) | Multifactor Primary Prevention Study | 7,495 | 45-56 | 30 | M | 451 | Hip fractures | Questionnaire | ICD-9, ICD-10 | age, height, body mass index, physical activity during leisure time, occupational class, smoking, coffee consumption, stroke before fracture and dementia before fracture |
| Sugiyama et al., 2010 (Japan) | CSRC | 292 | 32.4 | 20 | F | 33 | Vertebral fractures | NA | X-rays | age, sex, body mass index, initial age, smoking, initial GC dose, cumulative GC dose, number of GC dose increases, and GC pulse therapy |
| Lee et al., 2010  (Korea) | Korean Health and Genome Study (KHGS) | 9,351 | 40-69 | 3.8 | M/F | 195 | Osteoporotic fractures | Questionnaire | Self-report | age |
| Pluijm et al., 2009 (The Netherlands) | Rotterdam Study (ERGO), Longitudinal Aging Study Amsterdam (LASA) | 4,919 | >60 | 6-8.9 | F | 451 | Fragility fractures | Interview | Classified by general practitioner and reviewed by a medical expert | age, weight, body mass index, prior fracture, family history of hip fracture, systemic corticosteroid use, use of a walking aid, current smoking, rheumatoid arthritis, self-reported vertebral fracture |
| Cauley et al., 2009  (USA) | Study of Osteoporotic Fractures (SOF) | 9,158 | ≥65 | 10.5 | F | 462 | Hip fractures | Questionnaire | Medical records | age, weight, height, history of fracture, maternal hip fracture, steroid use, ever pregnant, surgical menopause, take walks for exercise, quadriceps, strength, walking speed, grip strength, near depth perception, contrast sensitivity, BMD |
| Moayyeri et al., 2009 (UK) | European prospective investigation into cancer (EPIC)-Norfolk study | 25311 | 40-79 | 10 | M/F | 925 | Any fractures | Questionnaire | ICD9, ICD10 | age, height, weight, history of fracture, BMI, smoking status, |
| Mukamal et al., 2007 (USA) | Cardiovascular Health Study (CHS) | 5,865 | ≥65 | 12 | M/F | 412 | Hip fractures | FFQ | Medical records | age, sex, race, current weight, height, smoking status, difficulty arising from a chair or bed, arthritis, diabetes, hypertension, clinical cardiovascular disease, previous cancer, weight in early teens, leisure-time physical activity, visual problems, MMSE score, and use of oestrogens, thiazide-type diuretics, thyroid agents, 15-foot walk time, falls, and self-reported health |
| Samelson et al., 2006 (USA) | Framingham Study | 704 | 54 | 25 | M/F | 135 | Vertebral fractures | Questionnaire | Radiographic examinations | age, sex, height, weight, smoking, physical activity, prevalent vertebral body fracture, relative metacarpal cortical area, duration of oestrogen use, and grip strength |
| Pluijm et al., 2006 (The Netherlands) | Longitudinal Aging Study Amsterdam (LASA) | 1,214 | ≥65 | 6 | M/F | 116 | Any fractures | Interview | Self-report | socio-demographic characteristics, chronic diseases and medication use, physical impairments and general health, body composition, activity and mobility, psycho-social functioning, current smoker, biochemical markers, other potential fall-related predictors |
| Cawthon et al., 2006 (USA) | Osteoporotic Fractures in Men (MrOS) study | 5,974 | ≥65 | 3.65 | M | 210 | Non-spine fractures | Interview | Physician review of radiology reports or study radiologist review of x-rays | age |
| Kanis et al., 2005  (The Netherlands, Australia and Canada) | Rotterdam Study, Dubbo Osteoporosis Epidemiology Study (DOES), Canadian Multicentre Osteoporosis Study (CaMos) | 16,971 | 65  (25-103) | 3-8 | M/F | 1,207 | Osteoporotic fractures | Interview | Self-report | current time, current age, alcohol intake, and alcohol intake times, current age, BMD, current smoking, body mass index |
|  |  |  |  |  |  | 279 | Hip fractures |  |  |  |
| Østbye et al., 2004 (Canada) | Canadian Study of Health and Aging (CSHA) | 5,570 | ≥65 | 5 | M/F | 355 | Any fractures | Interview | ICD-9 | age, stroke, heart disease, arthritis, inadequate vision, hormone replacement therapy, and number of children |
| Roy et al., 2003  (Europe) | European Vertebral Osteoporosis Study (EVOS) | 6,575 | 50-79 | 3.8 | M/F | 224 | Vertebral fractures | Questionnaire | Radiographic assessment | age and center |
| Siris et al., 2001  (USA) | The National Osteoporosis Risk Assessment | 163,979 | 64.5  (50-104) | 1 | F | NA | Osteoporotic fractures | Questionnaire | Self-report | age, body mass index site/device, race/ethnicity, prior fracture and oestrogen use |
| Hansen et al., 2000 (USA) | Iowa Women's Health Study | 34,703 | 61.6  (55-69) | 6.5 | F | 4,378 | Any fractures | FFQ | Postal surveys | age, coffee, calcium, oestrogen, smoking, activity, body mass index, calories, WHR |
|  |  |  |  |  |  | 1,128 | Wrist fractures |  |  |  |
|  |  |  |  |  |  | 275 | Hip fractures |  |  |  |
|  |  |  |  |  |  | 416 | Vertebral fractures |  |  |  |
|  |  |  |  |  |  | 288 | Forearm fractures |  |  |  |
|  |  |  |  |  |  | 389 | Upper arm fractures |  |  |  |
| Hoidrup et al., 1999 (Denmark) | Formerly the Glostrup Population Study, the Copenhagen City Heart Study, and the Copenhagen Male Study | 31,785 | 50.5  (20-93) | 13.6 | M/F | 807 | Hip fractures | Questionnaire | Medical records | age, study of origin, tobacco smoking, physical activity, body mass index, and school education |
| Bohannon et al., 1999 (USA) | Epidemiologic Studies of the Elderly (EPESE) | 2,590 | ≥65 | 6 | F | 540 | Any fractures | Interview | Self-report | race, age, education, family income, residence, underweight at age 50 years, overweight at age 50 years, tobacco use, no. of children, health, current medications |
|  |  |  |  |  |  | 438 | Non-hip, nonvertebral fractures |  |  |  |
| Mussolino et al., 1998 (USA) | First National Health and Nutrition Examination Survey | 2,879 | 45-74 | 22 | M | 71 | Hip fractures | Self-report | Hospital records and death certificates | Body mass index, previous fracture, current smoker, low nonrecreational physical activity, chronic conditions and phalangeal bone density |
| Jacqmin-Gadda et al., 1998 (France) | Paquid cohort study | 3,216 | ≥65 | 5 | M/F | 67 | Hip fractures | Interview | Self-report, confirmed by the physician | age, body mass index, sex, tobacco consumption, wine consumption, participation in sports, profession, visual or auditory impairment, use of the four classes of psychotropic drugs, and consumption of more than three non-psychotropics drugs |
|  |  |  |  |  |  | 238 | Non-hip fractures |  |  |  |
| Fujiwara et al., 1997 (Japan) | Adult Health Study (AHS) | 4,573 | 58.5 | 14 | M/F | 55 | Hip fractures | Questionnaire | Medical charts and radiographs | sex, age, body mass index, milk intake, prevalent vertebral fracture, follow-up period, along with age at menarche, number of children for women |
| Tuppurainen et al., 1995 (Finland) | Kuopio Osteoporosis Risk Factor and Prevention Study | 3,140 | 53.4 | 2.4 | F | 157 | Any fractures | Questionnaire | Self-report, verified by medical records | age |
|  |  |  |  |  |  | 42 | Wrist fractures |  |  |  |
| Cummings et al., 1995 (USA) | NA | 9,516 | ≥65 | 4.1 | F | 192 | Hip fractures | Questionnaire and Interview | Self-report, confirmed by reviewing radiographs | health status, ability to rise from chair |
| Hemenway et al., 1994 (USA) | Health Professionals Follow-up Study | 51,529 | 40-75 | 6 | M | 271 | Wrist fractures | Questionnaire | Self-report, validated by medical records | age, height, body mass index, smoking status |
| Hernandez-Avila et al., 1991 (USA) | Nurse' Health Study | 84,484 | 34-59 | 6 | F | 593 | Forearm fractures | FFQ | Self-report and medical records | age, Quetelet Index, calcium intake, menopause status, oestrogen-replacement therapy |
| Paganini-Hill et al., 1991 (USA) | Leisure World Study | 13,649 | 73 | 7 | M/F | 418 | Hip fractures | Questionnaire | Medical records | age |

^1^BMD, Bone mineral density; DHQ, diet history questionnaire; F, female; FFQ, food frequency questionnaire; ICD, international classification of diseases; M, male; NA, not available; *including: Greece, Germany, Italy, Norway, the Netherlands, Sweden, Spain, and the UK

**Supplemental Table 4** Quality assessment of included cohort studies (Newcastle-Ottawa Scale)

| Author (year) | a | b | c | d | e | f | g | h | i | Total |
| --- | --- | --- | --- | --- | --- | --- | --- | --- | --- | --- |
| Baleanu et al. (2022) | 1 | 1 | 0 | 1 | 1 | 1 | 1 | 1 | 1 | 8 |
| Rogmark et al. (2021) | 1 | 1 | 0 | 1 | 1 | 1 | 1 | 1 | 0 | 7 |
| Wang et al. (2020) | 1 | 1 | 0 | 1 | 1 | 1 | 1 | 0 | 1 | 7 |
| Swayambunathan et al. (2020) | 1 | 1 | 0 | 1 | 1 | 1 | 1 | 1 | 0 | 7 |
| Prieto-Alhambra et al. (2020) | 1 | 0 | 1 | 1 | 0 | 1 | 1 | 1 | 1 | 7 |
| Fung et al. (2019) | 1 | 1 | 1 | 1 | 1 | 1 | 0 | 1 | 1 | 8 |
| Wright et al. (2018) | 1 | 1 | 0 | 1 | 1 | 1 | 1 | 1 | 1 | 8 |
| Søgaard et al. (2018) | 1 | 1 | 1 | 1 | 1 | 1 | 1 | 1 | 0 | 8 |
| Saitz et al. (2018) | 0 | 1 | 1 | 1 | 1 | 1 | 0 | 0 | 0 | 5 |
| Kim et al. (2016) | 1 | 1 | 1 | 1 | 1 | 1 | 1 | 1 | 0 | 8 |
| Cauley et al. (2016) | 1 | 1 | 0 | 1 | 1 | 0 | 0 | 1 | 1 | 6 |
| Van der Veer et al. (2014) | 0 | 1 | 0 | 1 | 1 | 0 | 1 | 1 | 1 | 6 |
| Khabit et al. (2014) | 0 | 1 | 1 | 1 | 1 | 0 | 0 | 1 | 1 | 6 |
| Kubo et al. (2013) | 1 | 1 | 1 | 1 | 1 | 1 | 1 | 0 | 1 | 8 |
| Feart et al. (2013) | 1 | 1 | 1 | 1 | 1 | 1 | 0 | 1 | 1 | 8 |
| Benetou et al. (2013) | 1 | 1 | 0 | 1 | 1 | 1 | 0 | 1 | 1 | 7 |
| Womack et al. (2013) | 0 | 1 | 0 | 1 | 1 | 1 | 1 | 1 | 0 | 6 |
| Hippisley-Cox wt al. (2012) | 1 | 1 | 0 | 1 | 1 | 1 | 1 | 1 | 0 | 7 |
| Wuermser et al. (2011) | 1 | 1 | 0 | 1 | 1 | 0 | 1 | 1 | 0 | 6 |
| Trimpou et al. (2010) | 1 | 1 | 0 | 1 | 1 | 1 | 1 | 1 | 0 | 7 |
| Sugiyama et al. (2010) | 0 | 1 | 0 | 1 | 1 | 1 | 1 | 1 | 0 | 6 |
| Lee et al. (2010) | 1 | 1 | 0 | 1 | 1 | 0 | 0 | 0 | 0 | 4 |
| Pluijm et al. (2009) | 1 | 1 | 0 | 1 | 1 | 1 | 1 | 1 | 0 | 7 |
| Cauley et al. (2009) | 1 | 1 | 0 | 1 | 1 | 0 | 1 | 1 | 0 | 6 |
| Moayyeri et al. (2009) | 1 | 1 | 0 | 1 | 1 | 1 | 1 | 1 | 1 | 8 |
| Mukamal et al. (2007) | 1 | 1 | 0 | 1 | 1 | 1 | 1 | 1 | 1 | 8 |
| Samelson et al. (2006) | 1 | 1 | 1 | 1 | 1 | 1 | 1 | 1 | 1 | 9 |
| Pluijm et al. (2006) | 1 | 1 | 0 | 1 | 1 | 1 | 0 | 1 | 1 | 7 |
| Cawthon et al. (2006) | 1 | 1 | 0 | 1 | 1 | 0 | 1 | 0 | 1 | 6 |
| Kanis et al. (2005) | 1 | 1 | 0 | 1 | 1 | 1 | 1 | 1 | 0 | 7 |
| Østbye et al. (2004) | 1 | 1 | 0 | 1 | 1 | 0 | 1 | 1 | 0 | 6 |
| Roy et al. (2003) | 1 | 1 | 0 | 1 | 1 | 0 | 1 | 0 | 0 | 5 |
| Siris et al. (2001) | 1 | 1 | 0 | 1 | 1 | 0 | 0 | 0 | 1 | 5 |
| Hansen et al. (2000) | 1 | 1 | 1 | 1 | 1 | 1 | 0 | 1 | 0 | 7 |
| Hoidrup et al. (1999) | 1 | 1 | 0 | 1 | 1 | 1 | 1 | 1 | 1 | 8 |
| Bohannon et al. (1999) | 1 | 1 | 0 | 1 | 1 | 1 | 0 | 1 | 1 | 7 |
| Mussolino et al. (1998) | 1 | 1 | 0 | 1 | 1 | 1 | 1 | 1 | 1 | 8 |
| Jacqmin-Gadda et al. (1998) | 1 | 1 | 0 | 1 | 1 | 1 | 1 | 1 | 1 | 8 |
| Fujiwara et al. (1997) | 1 | 1 | 0 | 1 | 1 | 0 | 1 | 1 | 1 | 7 |
| Tuppurainen et al. (1995) | 1 | 1 | 0 | 1 | 1 | 0 | 1 | 0 | 1 | 6 |
| Cummings et al. (1995) | 1 | 1 | 0 | 1 | 1 | 0 | 1 | 0 | 1 | 6 |
| Hemenway et al. (1994) | 1 | 1 | 0 | 1 | 1 | 1 | 1 | 1 | 1 | 8 |
| Paganini-Hill et al. (1991) | 1 | 1 | 0 | 1 | 1 | 0 | 1 | 1 | 1 | 7 |
| Hernandez-Avila et al. (1991) | 1 | 1 | 0 | 1 | 1 | 0 | 1 | 1 | 1 | 7 |

a Representativeness of the exposed cohort

b Selection of the non-exposed cohort

c Ascertainment of exposure

d Demonstration that outcome of interest was not present at start of study

e Comparability of cohorts on the basis of the design or analysis (adjusted for age)

f Comparability of cohorts on the basis of the design or analysis (adjusted for smoking)

g Assessment of outcome

h Was follow-up long enough for outcomes to occur

i Adequacy of follow-up of cohort

**Supplemental Table 5** Subgroup analyses of dose–response risk of hip fractures with alcohol consumption

| Subgroup | Hip fractures (per 14 g/d increment) | | | | |
| --- | --- | --- | --- | --- | --- |
|  | No. of studies | RR (95%CI) | *I^2^*(%) | *P*^1^ | *P*^2^ |
| All studies | 18 | 1.04 (0.99-1.10) | 84.4 | < 0.001 |  |
| **Sex** |  |  |  |  | 0.286 |
| Male | 6 | 1.07 (0.96-1.18) | 88.6 | < 0.001 |  |
| Female | 8 | 1.05 (0.97-1.13) | 70.1 | 0.001 |  |
| Both | 4 | 0.98 (0.92-1.05) | 23.0 | 0.273 |  |
| **Age** |  |  |  |  | 0.950 |
| < 60 | 9 | 1.04 (0.97-1.12) | 89.1 | < 0.001 |  |
| ≥ 60 | 9 | 1.05 (0.95-1.15) | 62.9 | 0.006 |  |
| **Region** |  |  |  |  | 0.019 |
| US | 7 | 0.94 (0.85-1.04) | 61.4 | 0.016 |  |
| Non-US | 11 | 1.09 (1.03-1.16) | 85.8 | < 0.001 |  |
| **Sample size** |  |  |  |  | 0.936 |
| < 10000 | 3 | 1.01 (0.74-1.37) | 38.7 | 0.196 |  |
| ≥ 10000 | 15 | 1.04 (0.99-1.11) | 86.8 | < 0.001 |  |
| **Follow-up years** |  |  |  |  | 0.815 |
| < 8 years | 7 | 1.03 (0.93-1.15) | 68.7 | 0.004 |  |
| ≥ 8 years | 11 | 1.05 (0.98-1.12) | 86.5 | < 0.001 |  |
| **Study quality** |  |  |  |  | 0.213 |
| high | 17 | 1.05 (0.99-1.11) | 85 | < 0.001 |  |
| medium | 1 | 0.75 (0.49-1.15) | - | - |  |
| **Adjustment** |  |  |  |  |  |
| **Education** |  |  |  |  | 0.218 |
| Yes | 6 | 1.10 (1.02-1.20) | 68.5 | 0.007 |  |
| No | 12 | 1.01 (0.95-1.08) | 84.8 | < 0.001 |  |
| **BMD** |  |  |  |  | 0.083 |
| Yes | 2 | 1.23 (1.06-1.43) | 29.2 | 0.234 |  |
| No | 16 | 1.03 (0.97-1.09) | 85.3 | < 0.001 |  |
| **Fracture history** |  |  |  |  | 0.936 |
| Yes | 4 | 1.04 (0.94-1.15) | 83.8 | < 0.001 |  |
| No | 14 | 1.05 (0.98-1.12) | 83.4 | < 0.001 |  |
| **Smoking** |  |  |  |  | 0.286 |
| Yes | 15 | 1.06 (0.99-1.12) | 86.6 | < 0.001 |  |
| No | 3 | 0.98 (0.87-1.09) | 13.2 | 0.316 |  |

^1^BMD: Bone mineral density; CI: confidence interval; RR: relative risk.

^2^*P*^1^: *P* value for heterogeneity within each subgroup. *P*^2^: *P* value for heterogeneity between subgroups with meta-regression analysis.

**Supplemental Table 6** GRADE evidence table for the association of alcohol consumption with the risk of fractures

| **Quality assessment** | | | | | | | **No of patients** | | **Effect** | | **Quality** | **Importance** |
| --- | --- | --- | --- | --- | --- | --- | --- | --- | --- | --- | --- | --- |
|  |  |  |  |  |  |  |  |  |  |  |  |  |
| **No of studies** | **Design** | **Risk of bias** | **Inconsistency** | **Indirectness** | **Imprecision** | **Other considerations** | **Participants** | **Case (event rate)** | **Relative (95% CI)** | **Absolute** |  |  |
| **Total fractures** | | | | | | | | | | | | |
| 24 | Observational studies | No serious | No serious^1^ | No serious | No serious | Dose-response gradient | 5115456 | 170903 | RR 1.06 (1.02 to 1.10) | - | ⊕⊕⊕O MODERATE | IMPORTANT |
|  |  |  |  |  |  |  |  | 3.34% |  | - |  |  |
| **Osteoporotic fractures** | | | | | | | | | | | | |
| 23 | Observational studies | No serious | No serious^1^ | No serious | No serious | Dose-response gradient | 5115222 | 79251 | RR 1.05 (1.01 to 1.10) | - | ⊕⊕⊕O MODERATE | IMPORTANT |
|  |  |  |  |  |  |  |  | 1.55% |  | - |  |  |
| **Hip fractures** | | | | | | | | | | | | |
| 18 | Observational studies | No serious | No serious^1^ | No serious | Serious^2^ | Dose-response gradient | 4978064 | 31924 | RR 1.04 (0.99 to 1.10) | - | ⊕⊕OO LOW | IMPORTANT |
|  |  |  |  |  |  |  |  | 0.6% |  | - |  |  |
| **Wrist fractures** | | | | | | | | | | | | |
| 3 | Observational studies | No serious | No serious | No serious | Serious^2^ | Dose-response gradient | 87667 | 1472- | RR 0.98 (0.85 to 1.14) | - | ⊕⊕OO LOW | IMPORTANT |
|  |  |  |  |  |  |  |  | 1.68% |  |  |  |  |
| **Vertebral fractures** | | | | | | | | | | | | |
| 4 | Observational studies | No serious | Serious^3^ | No serious | Serious^2^ | Dose-response gradient | 36842 | 4556 | RR 1.34 (0.62 to 2.87) | - | ⊕OOO Very LOW | IMPORTANT |
|  |  |  |  |  |  |  |  | 12.3% |  |  |  |  |

^1^Substantial heterogeneity that was explained by the subgroup analyses and meta-regression analyses. Not downgraded.

^2^The 95% CI includes the null value (RR:1.00) and the upper bound of the 95% CI was ≥ 1.10. Downgraded.

^3^Substantial heterogeneity that was not explained by the subgroup analyses and meta-regression analyses. Downgraded.

**
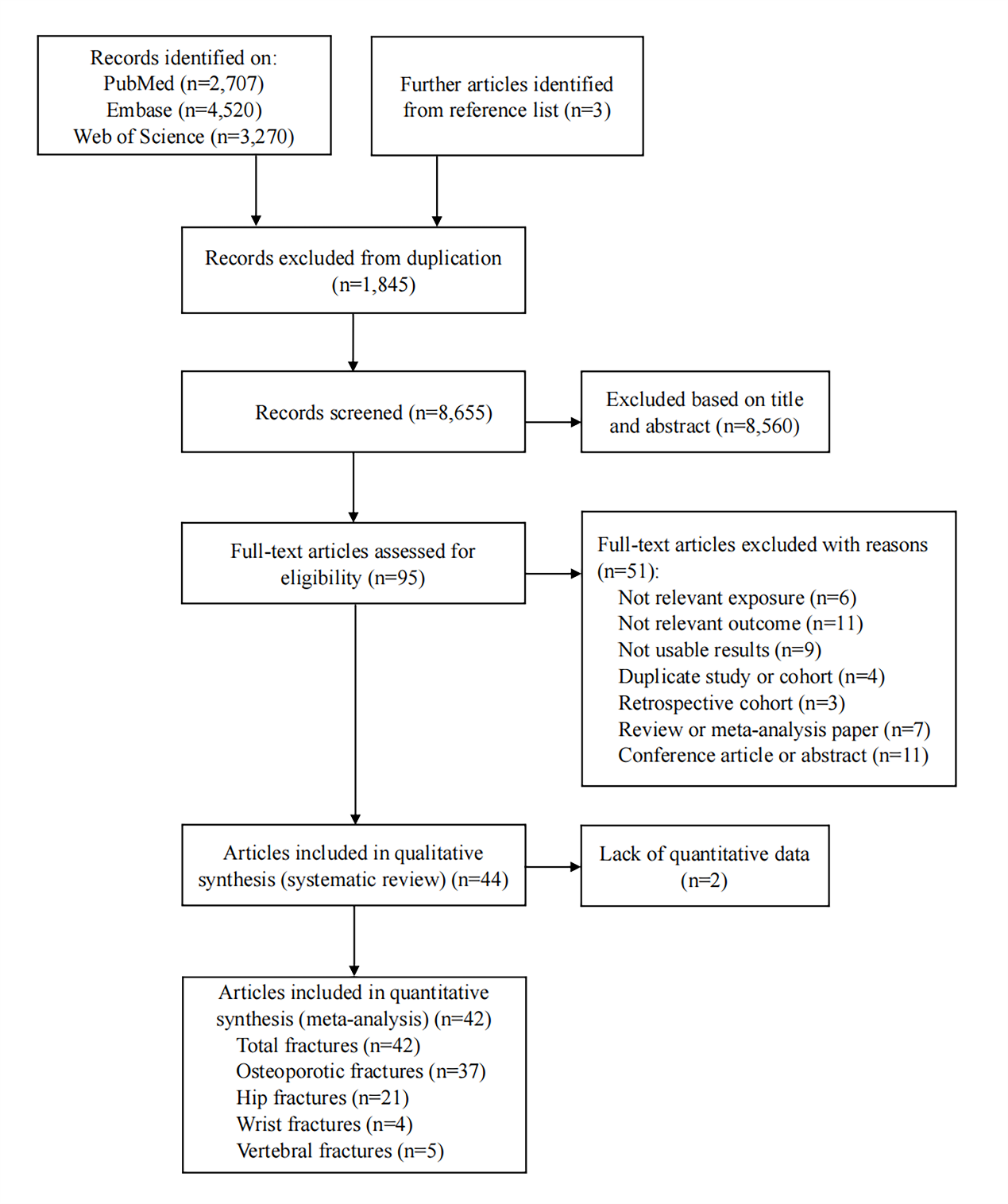
**

**Supplemental Figure 1** Flow–chart of article selection


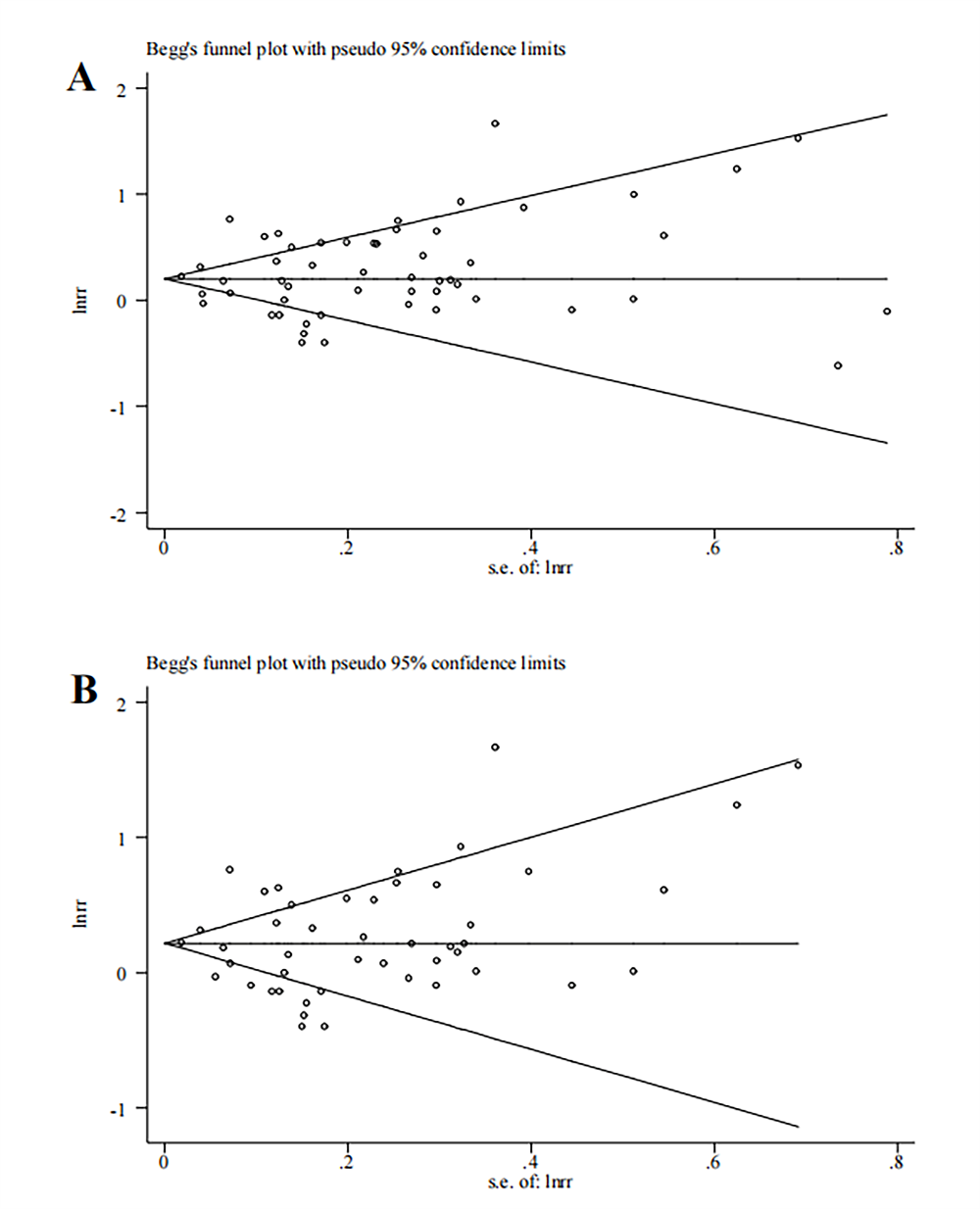


**Supplemental Figure 2** Funnel plot for publication bias for risk of fractures with highest versus lowest alcohol consumption level (A: total fractures, B: osteoporotic fractures)

**
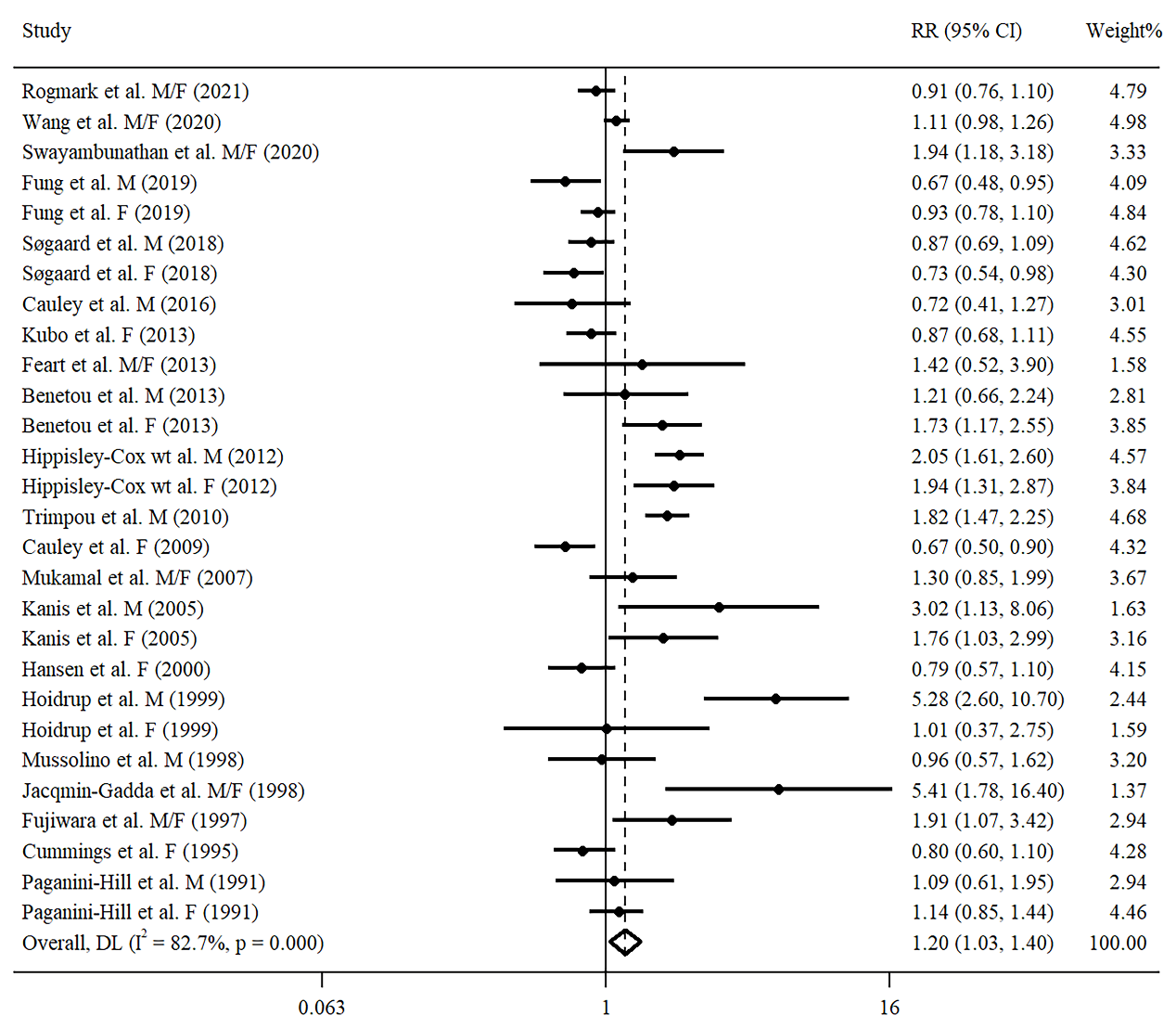
**

**Supplemental Figure 3** Forest plot of pooled relative risk for hip fractures with the highest versus lowest alcohol consumption level (CI, confidence interval; F, female; M, male; RR, relative risk)

**
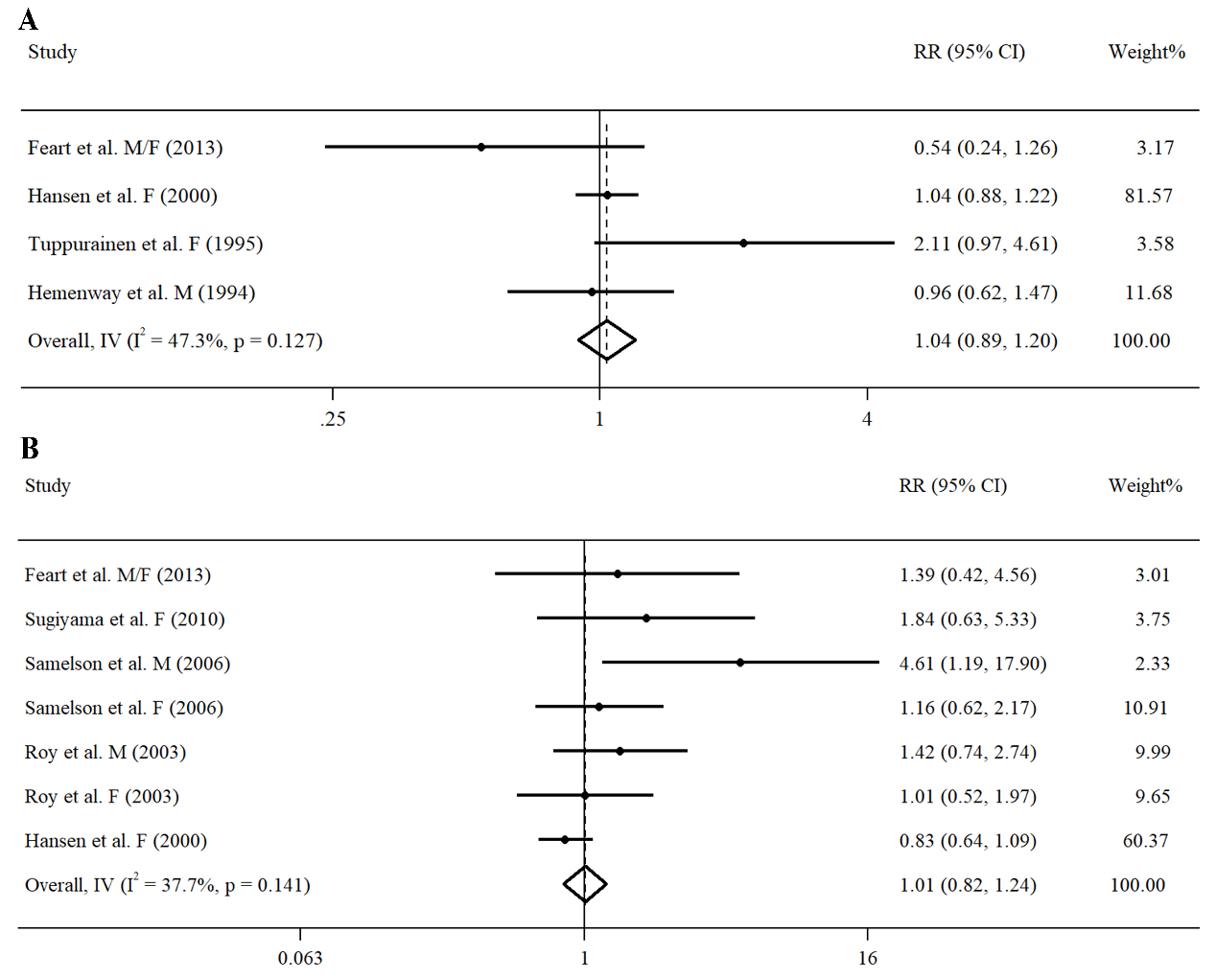
**

**Supplemental Figure 4** Forest plot of pooled relative risks for wrist fractures (A) and vertebral fractures (B) with the highest versus lowest alcohol consumption level (CI, confidence interval; F, female; M, male; RR, relative risk)


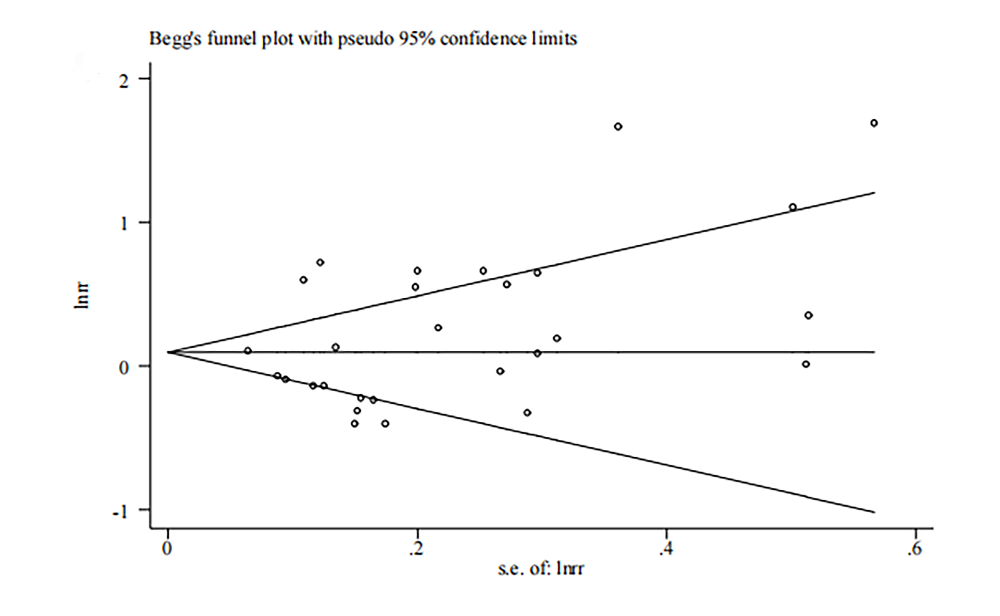


**Supplemental Figure 5** Funnel plot for publication bias for risk of hip fractures with highest versus lowest alcohol consumption level


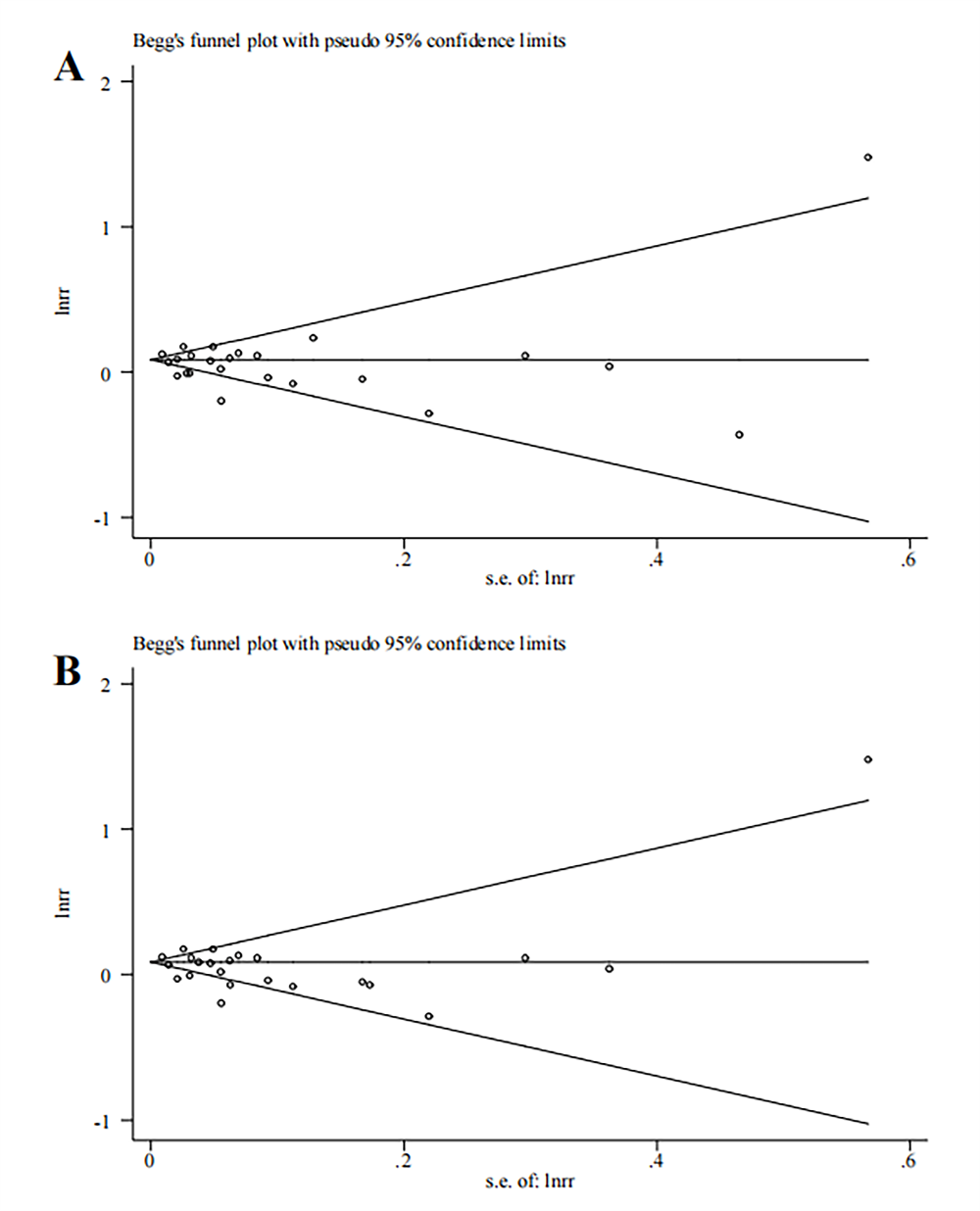


**Supplemental Figure 6** Funnel plot for publication bias for risk of fractures with per 14 g/d increment in alcohol consumption (A: total fractures, B: osteoporotic fractures)

**
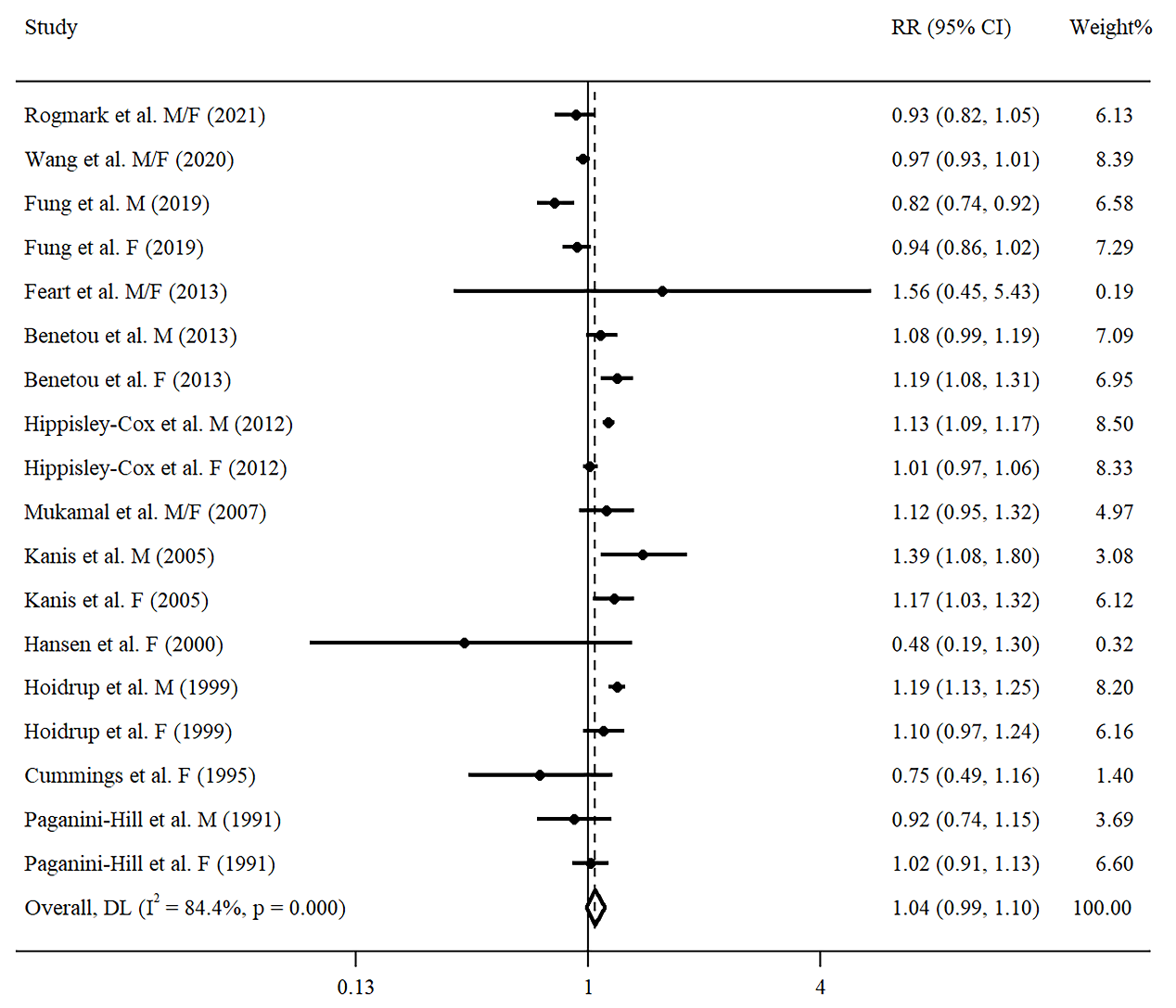
**

**Supplemental Figure 7** Forest plot of study-specific relative risk for hip fractures per 14 g/d increment in alcohol consumption (CI, confidence interval; F, female; M, male; RR, relative risk)


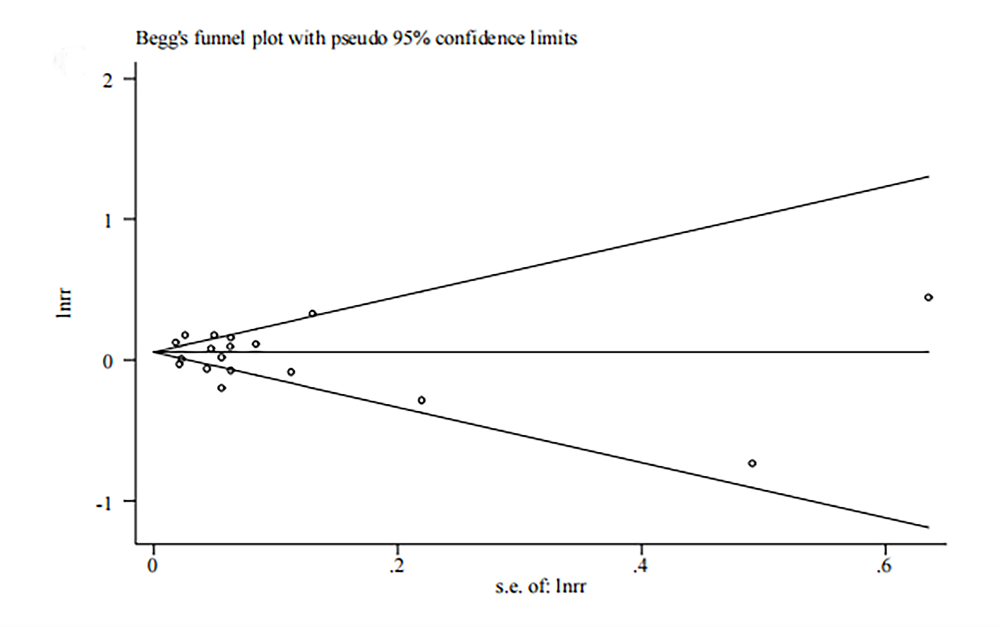


**Supplemental Figure 8** Funnel plot for publication bias for risk of hip fractures with per 14 g/d increment in alcohol consumption

**
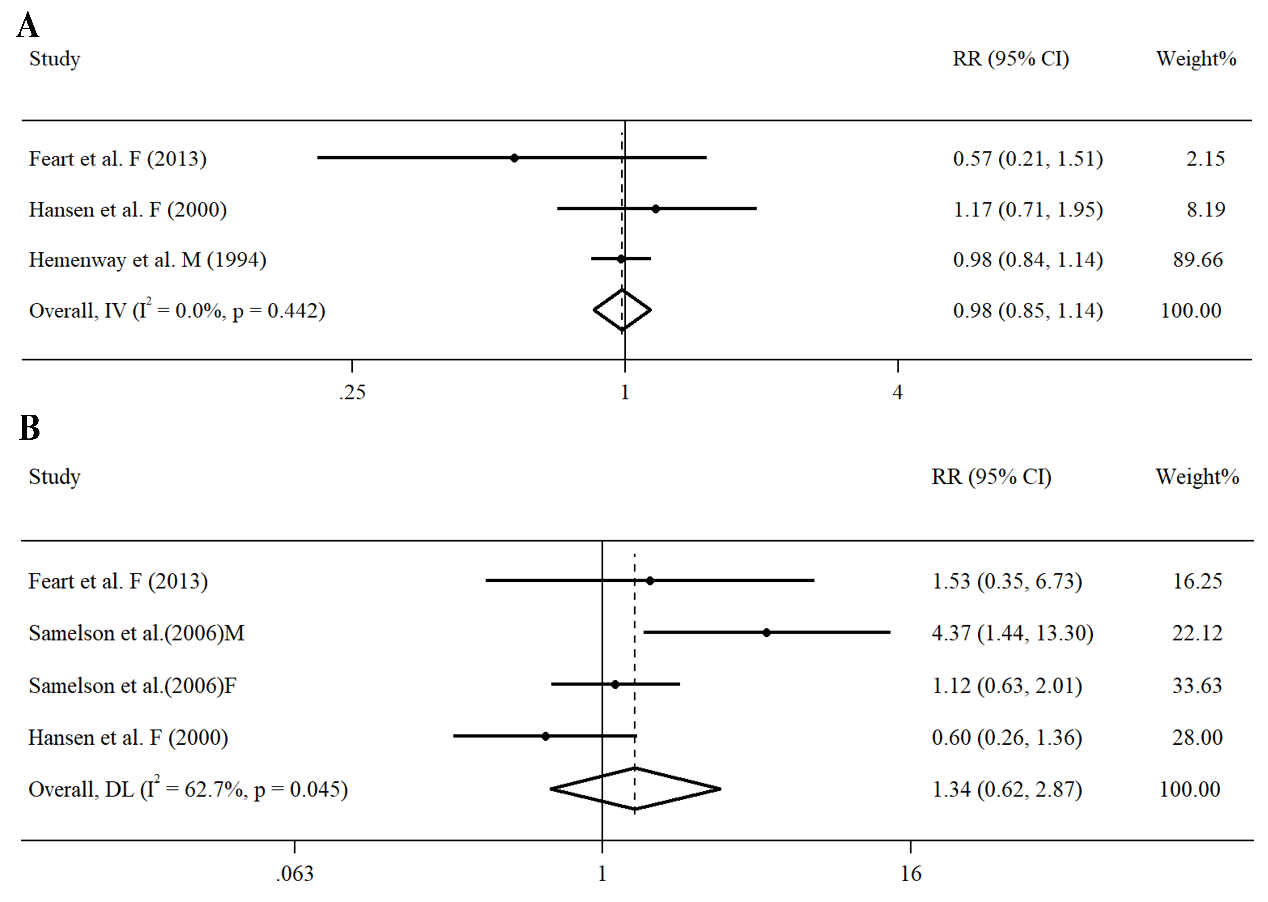
**

**Supplemental Figure 9** Forest plot of study-specific relative risks for wrist fractures (A) and vertebral fractures (B) per 14 g/d increment in alcohol consumption (CI, confidence interval; F, female; M, male; RR, relative risk)
